# Antioxidant Properties and Bioactive Compounds of Oregano, Sage, Basil, Rosemary, and Herbal Mixtures

**DOI:** 10.3390/antiox15091113

**Published:** 2026-09-04

**Authors:** Julia Płatkiewicz, Joanna Wróbel, Magdalena Jeszka-Skowron, Robert Frankowski, Zuzanna Grześkowiak, Beata Czarczyńska-Goślińska, Anna Maria Jeszka, Agnieszka Zgoła-Grześkowiak

**Affiliations:** 1 Institute of Chemistry and Technical Electrochemistry, Poznan University of Technology, Berdychowo 4, 60-965 Poznan, Poland; 2 Chair and Department of Pharmaceutical Technology, Poznan University of Medical Sciences, Rokietnicka 3, 60-806 Poznan, Poland; 3 Institute of Socio-Economics, Poznan University of Economics and Business, Al. Niepodległości 10, 61-875 Poznan, Poland

**Keywords:** herbs, oregano, sage, basil, rosemary, bioactive compounds, phenolic compounds

## Abstract

Herbs and spices are traditionally added to food in cuisines around the world. Antioxidant activity and content of bioactive compounds were compared in oregano, sage, basil, rosemary, and herbal mixtures. After optimization of ultrasound-assisted extraction of the ethanol–water extracts, the content of reducing compounds of the herb extracts was tested using the Folin–Ciocalteu method, and antioxidant capacity was evaluated with ABTS (2,2’-azinobis-(3-ethylbenzothiazoline-6-sulfonic acid)diammonium salt) and DPPH (2,2-diphenyl-1-picrylhydrazyl radical) assays. Oregano showed the highest antioxidant activity in all tests used (2.6 mg of gallic acid equivalent (GAE) per mL in the Folin–Ciocalteu test, 4.6 mg Trolox/mL in the ABTS assay, and 3.3 mg Trolox/mL in the DPPH assay) while rosemary had the lowest antioxidant activity (1.2 mg GAE/mL (Folin–Ciocalteu), 1.5 mg Trolox/mL (ABTS), and 1.3 mg Trolox/mL (DPPH)). Apart from antioxidant properties, the content of bioactive compounds was determined with the use of high-performance liquid chromatography–tandem mass spectrometry (LC-MS/MS). It was found that in all tested *Lamiaceae* herbs and the herbal mixes, rosmarinic acid widely predominates as a major non-volatile phenolic constituent, and its content varies from 1121 µg/g in rosemary to 10,255 µg/g in herbes de Provence. High concentrations were also observed for quinic acid in both oregano and rosemary. Interestingly, the concentrations of rosmarinic acid in the group of herbs studied are positively correlated with the results obtained in the Folin–Ciocalteu, ABTS, and DPPH tests (Spearman’s correlation coefficient 0.7030, 0.6657, and 0.7188, respectively), whereas no such correlation is observed for quinic acid. Overall, the findings indicate that these herbs share a common hydroxycinnamate-based phytochemical framework but display clear species-specific differences reflecting their intrinsic metabolism. Furthermore, the concentration of 3-caffeoylquinic acid in the Sicilian herbs (1021 µg/g) was approximately 10 times higher compared to the samples of Dalmatian herbs, herbes de Provence, and pure herbs, which demonstrates the unique chemical composition of that mixture, including the presence of dried tomatoes and tarragon, which were not included in other tested herbal mixtures.

## 1. Introduction

Spices and culinary herbs are widely used worldwide as flavoring agents and aromatic ingredients in a variety of foods, including salads, soups, pasta, meat, fish, vegetables, desserts, beverages, and liqueurs. In addition to their sensory properties, many herbs are recognized as important sources of bioactive phytochemicals, particularly phenolic acids and flavonoids, which contribute to their antioxidant potential. Among culinary herbs, species belonging to the *Lamiaceae* family are of particular interest because they are commonly consumed and are rich in phenolic constituents with potential health-promoting and food-preservative properties [1,2].

Oregano (*Origanum vulgare* L.), sage (*Salvia officinalis* L.), basil (*Ocimum basilicum* L.), and rosemary (*Rosmarinus officinalis* L.) are among the most widely used *Lamiaceae* herbs in fresh, dried, and processed forms, including pastes, sauces, marinades, soup mixes, flavored oils, and commercial herb mixtures. Although these herbs differ in botanical characteristics, sensory profiles, and culinary applications, they share a common feature: their biological activity is strongly associated with the presence of phenolic compounds. Previous studies have reported that oregano, sage, basil, and rosemary contain various phenolic acids and flavonoids, including rosmarinic acid, caffeic acid derivatives, chlorogenic acid, rutin, luteolin, apigenin, quercetin derivatives, and other polyphenolic constituents [3,4]. These compounds are considered major contributors to antioxidant activity and may also support the use of these herbs as natural preservatives in food systems.

Among the investigated herbs, oregano is frequently reported to contain high levels of phenolic compounds, including rosmarinic acid, flavonoids, and phenolic acid derivatives, which have been associated with strong antioxidant and antimicrobial properties [3,5,6,7]. Sage is also rich in phenolic constituents, particularly rosmarinic acid, salvianolic acid, epicatechin, and luteolin derivatives, and has been described as a source of compounds with antioxidant and preservative potential [3,8,9]. Basil contains a diverse profile of bioactive substances, including flavonoids, tannins, saponins, quinones, and volatile constituents, which may contribute to its antioxidant capacity [4,10,11]. Rosemary is well known for its high content of antioxidant compounds, especially rosmarinic acid and other phenolic diterpenes, and is widely recognized for its use as a natural antioxidant and preservative [3,12,13].

Despite the extensive literature describing the biological and pharmacological activities of these herbs, many previous studies focus on individual species, selected extracts, or essential oils rather than providing a systematic comparison of commercially available herbs and spice mixtures under the same extraction and analytical conditions. Moreover, the relationship between optimized extraction parameters, antioxidant activity, and the qualitative and quantitative profile of phenolic compounds remains insufficiently characterized for products available to consumers. This is particularly relevant because commercial herbs and herb mixtures may vary considerably depending on plant material, processing, supplier, and formulation, which can influence their antioxidant potential and phenolic composition.

Ultrasound-assisted extraction (UAE) was applied in this study as an efficient and relatively mild technique for obtaining herb extracts suitable for antioxidant and phytochemical analysis. The effectiveness of UAE is mainly attributed to acoustic cavitation, which promotes solvent penetration into plant tissues, disrupts cellular structures, and enhances mass transfer, thereby facilitating the release of bioactive constituents. Compared to conventional extraction approaches, UAE can reduce extraction time and improve the recovery of phenolic compounds and antioxidant-active substances. This is particularly relevant for herbs from the *Lamiaceae* family, which are rich in rosmarinic acid and other polar phenolics that are efficiently extracted using hydroethanolic solvents. Previous research on *Origanum vulgare* L. demonstrated that ultrasound pretreatment increased both total phenolic yield and antioxidant activity by approximately 36% compared with control extraction, especially when hydroethanolic solvents were used [14]. Therefore, UAE was selected as an appropriate extraction strategy for the studied herbs, enabling efficient recovery of phenolic antioxidants under controlled conditions.

Therefore, the aim of this study was to evaluate oregano, sage, basil, rosemary, and three commercially available spice mixtures (Sicilian herbs, Dalmatian herbs, and herbes de Provence) with respect to their antioxidant properties and phenolic composition. Ultrasound-assisted extraction was optimized using response surface methodology, and the obtained extracts were analyzed for antioxidant activity and phenolic compounds. This approach allowed a comparative assessment of individual herbs and commercial mixtures and provided insight into the relationship between extraction conditions, phenolic constituents, and antioxidant potential.

## 2. Materials and Methods

### 2.1. Chemicals

The analytical standards (Table 1) were obtained from Merck (Steinheim, Germany). All reagents used for LC-MS/MS analysis, including MS-grade acetonitrile, methanol, and formic acid, as well as those employed in radical scavenging assays—such as 2,2′-azino-bis(3-ethylbenzothiazoline-6-sulfonic acid) diammonium salt (ABTS), 2,2-diphenyl-1-picrylhydrazyl (DPPH), Folin–Ciocalteu reagent, Trolox, and gallic acid—were also obtained from Merck. Throughout the study, high-purity water of 18 MΩ·cm resistivity was used—prepared by reverse osmosis coupled with ion exchange purification (DEMIWA 5 ROSA, Watek, Ledeč nad Sázavou, Czech Republic) and then doubly distilled (quartz apparatus Bi18, Heraeus, Hanau, Germany).

### 2.2. Materials

All studied herbs are from the same family (*Lamiaceae* or *Labiatae*) and were purchased in bazaars and markets in Poznan, Poland. Detailed information describing the samples is given in Table 2. Before analysis, all samples were homogenized. For this purpose, all materials were ground for 1 min in a high-speed blender equipped with a stainless steel jar (model A11 basic; IKA Works GmbH and Co., Staufen, Germany).

### 2.3. Methods

For optimization of the extraction method, four herbs were used: oregano (O1), sage (S1), basil (B1), and rosemary (R1). A central composite design with two variables was applied: ethanol concentration in the aqueous mixture and time of ultrasound-assisted extraction. A 1.000 g sample of milled material was extracted with 20 mL of a solvent mixture (containing 0–100% ethanol), and the time of ultrasound treatment ranged from 5 to 30 min. The experimental parameters used in the optimization of herb extraction are provided in the Supplementary Materials in Table S1. The optimization was performed to obtain the best extraction conditions for the highest antioxidant potential and total phenolic content in the extracts.

The antioxidant potential and total phenolic content of the plant extracts were assessed using three spectrophotometric methods: the Folin–Ciocalteu assay, ABTS radical cation decolorization assay, and DPPH free radical scavenging assay. All analyses were carried out under dark conditions.

#### 2.3.1. Spectrophotometric Determination of Antioxidant Activity and Total Phenolic Content

The total phenolic content was determined using the Folin–Ciocalteu reagent, following the procedure described by Jeszka–Skowron and Zgoła–Grześkowiak [15]. Gallic acid served as the calibration standard, and the results were expressed as milligrams of gallic acid equivalents (GAE) per milliliter of extract.

The antioxidant capacity of the extracts was evaluated using the ABTS assay, as outlined by Re et al. [16]. Trolox was used as the reference antioxidant, and the outcomes were expressed as milligrams of Trolox equivalents (TE) per milliliter of extract.

The free radical scavenging activity was further assessed using the DPPH method, based on the procedure described by Jeszka–Skowron et al. [17]. The results were also presented as milligrams of Trolox equivalents (TE) per milliliter of extract.

#### 2.3.2. LC–MS/MS Determination of Bioactive Compounds

The identification and quantification of bioactive compounds were carried out using an UltiMate 3000 RSLC system (Dionex, Sunnyvale, CA, USA) coupled to an API 4000 QTRAP triple quadrupole mass spectrometer (AB Sciex, Foster City, CA, USA). A 5 µL aliquot of each sample was injected onto an Ascentis Express 90Å RP-Amide column (100 mm × 2.1 mm i.d., 2.7 µm; Merck), maintained at a constant temperature of 35 °C. Chromatographic separation was performed using a binary solvent system consisting of 0.1% formic acid in water (mobile phase A) and acetonitrile (mobile phase B), at a flow rate of 0.3 mL/min. The gradient elution program was as follows: 0 min—7% B, 2 min—15% B, 5 min—40% B, 6 min—60% B, 6.5 min—100% B, held until 8.6 min. The column effluent was directed to the electrospray ionization (ESI) source of the mass spectrometer, which was operated in both positive and negative ionization modes. The source parameters were set as follows: curtain gas 20 psi, nebulizer gas 45 psi, auxiliary gas 45 psi, source temperature 450 °C, ESI voltage ± 4500 V, and collision gas medium. Data acquisition was conducted in multiple reaction monitoring (MRM) mode with a dwell time of 50 ms per transition, using a scheduled MRM setup to ensure an adequate number of data points for each analyte.

Compound-specific detection parameters are detailed in the Supplementary Table S2. Additional LC–MS/MS parameters, including retention times, limits of detection (LOD), and limits of quantification (LOQ), are provided in the Supplementary Table S3. Representative chromatograms are given in Figure S1.

### 2.4. Statistical Analysis

The results were presented as mean values accompanied by standard deviations, based on a minimum of three independent replicates. Experimental optimization was conducted using a central composite design (CCD), comprising four factorial points, four axial points, and five center points. Model adequacy was assessed through analysis of variance (ANOVA), including an evaluation of lack of fit, the coefficient of determination (R^2^), adjusted R^2^, and the F-value obtained from the Fisher test. Statistical analyses were performed using the Statistica software, version 13.0 (StatSoft Inc., Tulsa, OK, USA).

The relationship between the content of the main active compounds present in the highest concentrations and the total phenolic content (Folin–Ciocalteu test results) or the antioxidant activity (ABTS and DPPH assays) was assessed using Spearman’s correlation. The calculations were carried out in Excel, taking into account both Spearman’s correlation coefficient and the *p*-value, which indicates whether the correlation is statistically significant or not.

## 3. Results and Discussion

### 3.1. Optimization of the Extraction Process of Herbs and Their Antioxidant Properties

The extraction process of herbal materials was systematically optimized using three analytical methods: the Folin–Ciocalteu assay and two radical-scavenging assays, ABTS and DPPH. Response Surface Methodology (RSM) was applied to model the extraction process, and the optimal parameters were selected based on the statistical significance of the results obtained from the analysis of variance (ANOVA). Among the evaluated variables, ultrasound treatment time and the composition of the solvent mixture were identified as the key factors influencing extraction efficiency. The applied optimization strategy made it possible to determine extraction conditions that maximized the antioxidant potential of the resulting solutions. The objective of this optimization was to establish the most favorable extraction conditions to ensure the highest recovery of bioactive and antioxidant compounds. The Supplementary Materials contain detailed results of the extraction process optimization for four herbs using three spectrophotometric tests (Tables S4–S7). Response surfaces showing changes in analytical signals as a function of extraction time and ethanol concentration for individual herbs in the Folin–Ciocalteu, ABTS, and DPPH tests are presented in Table S8. The Pareto charts presenting the statistical significance of extraction time and ethanol content, including linear (L) and quadratic (Q) terms for individual herbs in the Folin–Ciocalteu, ABTS, and DPPH tests, are shown in Table S9. The vertical red line on each chart indicates the boundary for statistical significance, corresponding to a 95% confidence level. Overall, the Pareto charts demonstrate that both studied factors significantly affect the extraction of bioactive compounds. A summary of the statistical data obtained as a result of the optimization for all the tests carried out is given in Tables S10–S21.

The results from the individual tests and herbs guided decisions regarding the extraction time and the percentage composition of the extraction mixture. Within a given herb, the results obtained for each test used were consistent (Table S22). Based on the optimization carried out, the largest amount of antioxidants from the herbs was transferred into the solution using a 30 min ultrasound-assisted extraction. The extraction mixture used for basil contained the lowest amount of ethanol, only 10%. In the case of oregano, this content was 50% of the organic phase, and for sage and rosemary, 55%. A 55% ethanol content was also used for herbal mixture samples. The tests were carried out on all herb samples under the specified conditions. Table 3 presents the results obtained for the herb samples. The heat maps were presented independently for each column, as the principle behind each of the three tests is different.

#### 3.1.1. Objective Presentation of Extraction Optimization and Antioxidant Results

The optimization results indicated that ultrasound-assisted extraction for 30 min provided the most favorable extraction conditions for all investigated herbs. The optimized ethanol concentration differed depending on the plant material: 10% ethanol was selected for basil, 50% ethanol for oregano, and 55% ethanol for sage and rosemary. The same 55% ethanol solution was subsequently used for the herbal mixture samples. Under these conditions, antioxidant-related responses were determined for all individual herbs and commercial herbal mixtures using the Folin–Ciocalteu, ABTS, and DPPH assays, and the numerical results are summarized in Table 3.

Among the individual herbs, oregano showed the highest values in all three assays, with sample O4 giving the highest response in the Folin–Ciocalteu, ABTS, and DPPH tests. Basil and sage showed intermediate activity, although the values varied among producers. Rosemary produced the lowest antioxidant responses among the pure herbs in all three assays. Among the herbal mixtures, the tested herbes de Provence samples showed variable results, while the Dalmatian and Sicilian herb mixtures displayed lower or intermediate activity depending on the assay applied.

#### 3.1.2. Interpretation of Antioxidant Responses and Extraction-Related Effects

The Folin–Ciocalteu test estimates total polyphenol content in herbal samples, although it measures their overall reducing potential rather than specific phenolics. As a result, the Folin–Ciocalteu reagent can be reduced by other reducing agents present in the extract, which may lead to an overestimation of phenolic compounds. Although this test allows the reducing potential of the sample to be assessed, it does not provide direct information on antioxidant activity. Therefore, the ABTS and DPPH assays were performed, which use the free radical scavenging ability of the bioactive compounds in the extract. As is known, the ABTS test works for both lipophilic and hydrophilic antioxidants, while the DPPH test works only for lipophilic antioxidants. The use of these two tests provides complementary information. The results showed that extracts containing higher concentrations of phenolic compounds generally exhibited higher activity in the ABTS and DPPH tests. Furthermore, the ABTS results were higher than the DPPH results, which may be due to the reaction of the ABTS^+^• cation radical with both hydrophilic and lipophilic antioxidants, or mainly due to the presence of hydrophilic antioxidants. Therefore, ABTS can better reflect the total radical-scavenging potential of extracts, while DPPH provides complementary information, especially about antioxidants that are more compatible with less polar reaction conditions. Consequently, the ABTS results were higher for herb extracts than the DPPH results, when hydrophilic phenolics contribute substantially to antioxidant activity.

Oregano showed by far the highest antioxidant activity in all tests used. This indicates that oregano extract has the highest content of reducing compounds, mainly polyphenols, and the greatest ability to scavenge both hydrophilic and lipophilic radicals. Oregano from Prymat had the highest content of bioactive compounds, approximately 1.5 times more than the other samples. This result is consistent with the available literature, which indicates that oregano is rich in phenolic compounds with strong antioxidant potential, particularly rosmarinic acid, caffeic acid, and various flavonoids such as luteolin and apigenin glycosides [18].

Studies on *Origanum vulgare* have identified rosmarinic acid as one of the dominant phenolics. These studies linked oregano extracts with high antioxidant activity in assays such as DPPH, ABTS, and FRAP (Ferric Reducing Ability of Plasma—used for measuring the total antioxidant activity of a sample), connecting this activity to its high phenolic density and favorable extractability in hydroalcoholic systems [18]. Furthermore, more recent work comparing advanced extraction techniques confirmed that oregano extracts obtained using ultrasound- and microwave-assisted methods exhibit high total phenolic content and strong radical-scavenging activity. These results support the present findings, indicating that oregano responds particularly well to intensified extraction processes [18,19,20].

Sage and basil showed similar levels of antioxidant activity, suggesting that the amount and/or activity of phenolic compounds and other antioxidants present in them are comparable. Although the extracts of both herbs showed similar responses in the ABTS and DPPH tests, it should be noted that the basil extract was prepared in a different solvent mixture (10% ethanol), which may have limited the extraction of more lipophilic antioxidants. Despite this, the results obtained for basil were similar to those of sage, indicating that its antioxidants are mostly hydrophilic or easily extracted in an aqueous environment. This interpretation is consistent with the known phytochemical profile of basil, in which polar phenolic constituents, including rosmarinic acid and other hydroxycinnamic acid derivatives, contribute substantially to antioxidant capacity. Sage, in turn, contains both polar phenolics and less polar diterpenes; therefore, its antioxidant behavior may depend more strongly on extraction conditions and solvent polarity. The lowest test results were obtained for sage from Belin. These contents were more than three times lower than in the other samples, which may indicate lower raw material quality or compositional degradation associated with processing or storage.

Rosemary showed the lowest antioxidant activity among the herbs analyzed, which is surprising given the high content of carnosol and carnosic acid—powerful antioxidant diterpenes characteristic of this plant—as reported in the literature [21,22]. This discrepancy should not be interpreted solely as a consequence of solvent polarity, although polarity is an important factor. Rather, the measured antioxidant response reflects the combined effects of solvent composition, extraction kinetics, plant matrix structure, analyte localization, compound stability, and the analytical selectivity of the applied assays. Carnosic acid and carnosol are relatively lipophilic and may be less efficiently released into hydroethanolic extracts than more polar phenolic acids, such as rosmarinic acid. At the same time, these diterpenes may be retained within glandular structures, resinous fractions, or less accessible plant tissues, meaning that even an optimized ultrasound-assisted extraction may recover only the extractable fraction available under the selected conditions. The literature confirms that rosemary extraction is strongly affected by ethanol concentration, temperature, time, matrix pretreatment, and the extraction technique applied; ultrasound can enhance mass transfer and tissue disruption, but it does not necessarily guarantee exhaustive recovery of all antioxidant constituents [21,23,24].

Therefore, the relatively low antioxidant activity observed for rosemary may indicate not only incomplete solubilization caused by solvent mismatch, but also incomplete depletion of antioxidant compounds from the plant residue. This is an important limitation of the present interpretation. A more comprehensive assessment would require supplementary experiments on the post-extraction residues, for example, by re-extracting the solid residue with a less polar solvent system, applying sequential extraction with solvents of different polarity, or directly determining residual antioxidant activity and residual carnosic acid and carnosol content in the solid residue remaining after extraction. Such analyses would make it possible to distinguish between genuinely low antioxidant potential of the tested rosemary samples and limited analytical recovery under the selected extraction conditions. In the absence of residue-based measurements, the present results should therefore be interpreted as the antioxidant activity of the obtained extracts rather than the total antioxidant capacity of the original rosemary material. Consequently, despite the use of an optimized method for extracting bioactive compounds, the results may not fully reflect the actual antioxidant reservoir of the biological material, but rather the portion effectively released and measurable under the applied experimental conditions.

The main research results concerning the effect of extraction solvent and procedure are consistent with broader literature showing that ultrasound-assisted extraction is an efficient alternative to conventional solvent extraction. Previous studies have demonstrated that UAE shortens extraction time, improves penetration of hydroalcoholic solvents into plant tissues, and increases the release of phenolic compounds through cavitation-induced disruption of cell walls and enhancement of mass transfer [24]. These mechanisms are particularly important when ethanol–water mixtures are used, since the combination of solvent polarity tuning and ultrasound energy can substantially improve the extraction of both total phenolics and antioxidant-active constituents. In this context, the present optimization results further confirm that both extraction time and ethanol concentration are critical parameters and that their interaction must be considered to maximize extract quality.

Various commercially available herbal blends were also tested to examine their antioxidant properties. These include herbs de Provence, which are aromatic mixtures typically consisting of rosemary, thyme, savory, marjoram, basil, and oregano, sometimes with the addition of lavender or sage. As these blends vary in their composition, the results obtained are inconsistent. The lowest concentrations in the tests were obtained for a 5-ingredient blend that did not declare the presence of oregano in its composition. This finding further supports the key contribution of oregano to the antioxidant potential of herbal mixtures. Given its consistently high phenolic content and strong radical-scavenging activity reported both in this study and in the literature, the presence or absence of oregano may substantially affect the overall antioxidant profile of commercial herb blends. At the same time, differences between blends may also arise from variability in the proportion of ingredients, botanical origin, and processing history, which should be taken into account when interpreting the antioxidant potential of mixed herbal products.

### 3.2. LC-MS/MS Analysis for Herbs from Different Producers

The herbal sample extracts were also analyzed using LC-MS/MS. The analytical method developed enabled the determination of 39 compounds. The results obtained are presented in Table 4. The heat maps were applied separately to each column in order to better highlight the relationship between the content of the compounds and the type of sample.

The results obtained in this study are generally in agreement with the scientific literature on *Lamiaceae* herbs. Previous studies identified rosmarinic acid as one of the dominant phenolic constituents in oregano, basil, rosemary, and sage, although its concentration may vary markedly with plant genotype, cultivation conditions, post-harvest processing, and extraction procedure. Furthermore, the literature indicates that oregano is often particularly rich in rosmarinic acid, which is consistent with the high levels observed here. In the present study, the highest concentrations in each herb and herbal mixture were found for rosmarinic acid and quinic acid. In oregano, both compounds occurred at similarly high levels (3600–9800 µg/g of ROSM and 4100–7900 µg/g of QUI), whereas in rosemary their concentrations were lower than in oregano but remained within a comparable order of magnitude (1100–9600 µg/g of ROSM and 1400–6100 µg/g of QUI). In basil, rosmarinic acid clearly predominated over quinic acid (3500–8200 µg/g of ROSM and 900–1600 µg/g of QUI), while in sage, quinic acid was present only at trace levels relative to rosmarinic acid (1000–15,600 µg/g of ROSM and 20–65 µg/g of QUI). This distribution is consistent with scientific literature describing rosmarinic acid as a principal phenolic acid in these herbs, while emphasizing that the relative abundance of quinic acid and its derivatives can be strongly matrix- and method-dependent [25].

De A.R. Oliveira et al. [26] investigated the efficiency of acetone, methanol, ethanol, and their aqueous mixtures for the quantitative extraction of rosmarinic acid, carnosol, and carnosic acid. Their results indicated that 59–70% ethanol and 80% acetone provided comparable extraction performance, whereas 50% methanol resulted in lower carnosic acid recovery, likely due to its conversion to carnosol. Based on these findings, the authors applied a central composite design to optimize the simultaneous extraction of the three target compounds using ethanol–water systems. The optimal conditions were identified as 70% ethanol, a solid-to-liquid ratio of 1:5, and an extraction time of 55 min, which enabled recovery of approximately 90% of the antioxidants while maintaining high extract purity. In separate maceration experiments using ethanol concentrations ranging from 30% to 96%, 50% ethanol yielded the highest total phenolic content and antioxidant activity [27].

Ethanol–water mixtures are widely regarded as green solvents and have therefore been adopted in related studies [28,29]. Similarly, Psarrou et al. [30] evaluated aqueous ethanol and acetone systems and reported that the use of 60% ethanol or 60% acetone resulted in the highest total phenolic content, antiradical activity, and extraction selectivity. Compared to pure water, aqueous organic solvent mixtures offer superior extraction efficiency because they facilitate the recovery of both less polar constituents, such as phenolic diterpenes and flavonoid aglycones, and more polar compounds, including phenolic acids and flavonoid glycosides. The same study also examined extraction kinetics and identified an initial rapid extraction phase followed by a slower stage, with both processes conforming to the unsteady-state diffusion model. Increasing the extraction temperature from 22 to 60 °C promoted matrix swelling, solute solubilization, and diffusion, thereby accelerating extraction. However, higher temperatures reduced selectivity due to the co-extraction of non-flavonoid compounds. Although total terpenoid recovery increased with temperature, a substantial proportion of carnosic acid was converted to carnosol at 60 °C [30].

Significant amounts of succinic acid were found in extracts of basil (397–847 µg/g) and sage (288–688 µg/g). A wide range of concentrations was reported for rosemary (68–461 µg/g). In contrast, SUC concentrations in oregano were relatively low (54–73 µg/g). Because succinic acid is less frequently discussed as a key discriminating phytochemical in review articles on these herbs, this observation should be interpreted more cautiously and considered mainly as a feature of the analyzed samples rather than a universally established chemotaxonomic trend. By contrast, carnosic acid and carnosol are mainly found in extracts obtained from sage (55–441 µg/g and 28–72 µg/g, respectively) and rosemary (15–546 µg/g and 23–110 µg/g, respectively), while in oregano and basil, they remain undetectable or are present in insignificant amounts. Their occurrence mainly in rosemary and sage agrees well with the literature, since these two herbs are known to accumulate phenolic diterpenes, especially carnosic acid and carnosol, at substantially higher levels than basil or oregano. Likewise, the finding that rutin was determined in considerable amounts mainly in basil (350–1230 µg/g) is plausible, although flavonoid composition is known to vary substantially among cultivars and analytical conditions [31,32]. The high content of rutin, which is relatively easily soluble in water, may also result in extraction conditions for basil that yield high antioxidant activity even at low ethanol concentrations, as demonstrated in this publication.

The results further indicate that rosemary and sage contain quantifiable amounts of caffeic, ferulic, and syringic acids, together with carnosol, which is in line with published phytochemical profiles of these herbs. At the same time, the absence of melatonin and the non-detection or trace occurrence of compounds such as theophylline, synapic acid, theobromine, myricetin, gallocatechin, catechin gallates, epicatechin, gallocatechin, and epigallocatechin should be interpreted with caution, as “not detected” reflects the applied analytical method and its detection limits rather than their definitive absence in the plant matrix. Sik et al. [33] explicitly note that sample preparation and analytical configuration can strongly influence the detectability of phenolic compounds in complex herbal matrices, and Zhang et al. [34] similarly emphasize the method dependence of qualitative and quantitative phytochemical outcomes. Therefore, absence in the analytical profile should be discussed conservatively as non-detection under the applied conditions, rather than as biochemical absence per se. Overall, the present results support the view that oregano, basil, rosemary, and sage are rich in phenolic constituents, with rosmarinic acid as a common major compound and rosemary/sage distinguished by a higher contribution of diterpenoid antioxidants such as carnosol [25,32].

The phytochemical profiles obtained in the present study are broadly consistent with the well-established compositional patterns reported for culinary herbs of the *Lamiaceae* family, in which rosmarinic acid is repeatedly identified as one of the dominant non-volatile phenolic constituents of oregano, basil, rosemary, and sage. Both Guan et al. [25] and Sik et al. [33] emphasize that rosmarinic acid is among the most characteristic phenolic acids in Nepetoideae taxa and frequently serves as a major contributor to their antioxidant potential. The predominance of rosmarinic acid across all four herbs examined here, therefore, agrees with the current consensus that hydroxycinnamic acid derivatives are central markers of *Lamiaceae* phytochemistry. This interpretation is further supported by broader surveys of the family, showing that rosmarinic acid and related phenolic acids commonly co-occur with high antioxidant activity across multiple aromatic species. Moshari–Nasirkandi et al. [35] reported that rosmarinic acid, together with chlorogenic-type derivatives, represented major phenolics in several *Lamiaceae* taxa, while Grigore–Gurgu et al. [31] likewise highlighted phenolic acids as core bioactive constituents of oregano, rosemary, sage, and basil.

At the same time, the marked quantitative differences observed among species are in line with the literature. Numerous studies have shown that the concentration of rosmarinic acid depends strongly on genotype, plant organ, developmental stage, environmental conditions, agronomic practice, post-harvest treatment, drying, and extraction methodology. Sik et al. [33] discuss the substantial analytical variability arising from extraction and chromatographic conditions, whereas Guan et al. [25] emphasize that biosynthesis and accumulation of rosmarinic acid are strongly modulated by both plant genetics and environmental cues. In basil specifically, cultivar and developmental stage have also been shown to influence phenolic composition, including rosmarinic and caffeic acid derivatives. Lawson et al. [36] demonstrated significant cultivar × maturity interactions for basil phenolics, with rosmarinic acid generally increasing during vegetative development in a genotype-dependent manner. Consequently, the variability observed in the present material should not be interpreted as anomalous, but rather as a predictable consequence of biological and methodological heterogeneity.

Among the analyzed herbs, the particularly high rosmarinic acid content in oregano is chemically credible and aligns well with previous reports identifying oregano as one of the richest culinary sources of phenolic antioxidants within the *Lamiaceae* family. Khorsand et al. [37] showed that oregano accessions differ widely in phenolic composition yet consistently display high abundance of phenolic constituents and strong antioxidant properties. Likewise, Bouloumpasi et al. [38] reported that oregano extracts retained strong antioxidant activity associated with their phenolic fraction. These reports support the interpretation that the high rosmarinic acid levels observed here in oregano are not only plausible, but are consistent with oregano’s recognized status as a phenolic-rich and functionally potent herb [37,38].

The coexistence of substantial quinic acid and rosmarinic acid in some matrices, especially oregano, is likewise plausible, although QUI is less consistently emphasized in herb-focused reviews than ROSM. From a biochemical perspective, QUI is a key intermediate in the shikimate/phenylpropanoid network, and esterified QUI derivatives (such as chlorogenic acids and related acyl-quinates) are widespread in plants. Marchiosi et al. [39] and Clifford et al. [40] underline the central role of QUI as a biochemical scaffold in plant phenolic metabolism. Thus, the comparatively high QUI levels detected in oregano and rosemary may reflect species-specific differences in carbon partitioning through hydroxycinnamate-related pathways, but they may also depend strongly on matrix composition and extraction selectivity. In this sense, QUI is best interpreted as a credible accessory component of the phenolic network rather than as a primary chemotaxonomic marker equivalent to ROSM.

The results for rosemary are also highly consistent with published phytochemical evidence. Rosemary is widely recognized as an herb containing both polar phenolic acids and lipophilic diterpenes, particularly carnosic acid and carnosol, with the relative abundance of these fractions being strongly dependent on extraction conditions. Lešnik et al. [41] provide a broad overview of this dual phytochemical character, while Loussouarn et al. [21] confirm the central role of diterpenes in rosemary antioxidant defense. Zhu et al. [42] further illustrate how rosmarinic acid and carnosic acid can be differentially enriched depending on separation strategy, reinforcing the view that rosemary extracts often occupy an intermediate compositional position between phenolic-acid-rich and diterpene-rich matrices. Therefore, the present rosemary profile, characterized not only by ROSM and QUI but also by the caffeic, ferulic, and syringic acids, and appreciable carnosol, is fully compatible with the established chemistry of the species.

A similar interpretation applies to sage, whose phytochemical pattern is generally regarded as overlapping with rosemary in the presence of phenolic diterpenes, though the plant still retains high levels of rosmarinic acid. Recent quantitative data on commercial dried sage reinforce this conclusion. Hrebień–Filisińska et al. [43] documented a very wide range of rosmarinic acid, carnosic acid, and carnosol concentrations among commercial samples, but consistently confirmed all three as key non-volatile bioactives. The observation in the present study that sage contained high rosmarinic acid together with detectable carnosol, while quinic acid remained only at a trace level, is therefore chemically credible and appears to reflect the characteristic specialization of sage toward antioxidant phenolics and diterpenoid antioxidants rather than toward high accumulation of simple organic acids. Moshari–Nasirkandi et al. [35] found that Salvia species tend to rank among parts of the *Lamiaceae* taxa that is richer in antioxidant-relevant phenolics.

The pattern observed for basil is likewise in agreement with the literature. Basil is widely recognized as a relevant source of soluble phenolics, particularly rosmarinic acid, while its flavonoid fraction may vary considerably according to cultivar and growing conditions. Kiferle et al. [44] and Lawson et al. [36] indicate that rosmarinic acid accumulation in basil is dynamic and highly dependent on tissue type and developmental stage. The comparatively high rutin content observed mainly in basil in the present study is also plausible because basil is known to accumulate flavonoids in cultivar-dependent amounts, and its phenolic composition is sensitive to agronomic and environmental variation. Pham et al. [45] further confirm that basil extracts can yield substantial levels of rosmarinic acid under suitable conditions. Collectively, these studies support the interpretation that the present basil profile—high rosmarinic acid, moderate quinic acid, and relatively elevated rutin—fits the concept of basil as a herb characterized mainly by phenolic acids and flavonoids rather than by the diterpenoid antioxidant signature typical of rosemary and sage.

From a methodological perspective, the quantitative differences among herbs should also be interpreted in the context of solvent polarity, extraction temperature, and extraction time, all of which are known to strongly influence the recovery and apparent relative abundance of plant secondary metabolites. Reviews of phenolic compound extraction consistently show that aqueous ethanol systems are often among the most effective “green” solvents for recovering phenolic acids from aromatic plants. Zhang et al. [34] and Irakli et al. [46] both indicate that ethanol concentration is one of the most influential factors affecting phenolic yield. In rosemary, Vieira et al. [47] demonstrated clearly that polar systems preferentially recover rosmarinic acid, whereas nonpolar media enrich carnosic acid and carnosol. This is highly relevant for interpreting the present findings, because the extract composition reflects not only botanical differences but also solvent-driven selectivity.

Temperature-related transformations are equally important. The literature indicates that thermal and oxidative conditions can alter the balance between carnosic acid and carnosol, since carnosol is a recognized oxidation product of carnosic acid [48]. Loussouarn et al. [21] provide mechanistic evidence that carnosic acid is consumed under oxidative conditions and converted into derivatives, including carnosol. Similar conclusions have been reported for sage matrices, where extraction conditions influence not only recovery but also compound stability. Hrebień–Filisińska et al. [43] showed that rosmarinic acid is generally more stable than carnosic acid, while the latter undergoes greater degradation and transformation depending on the solvent system. Accordingly, the presence of carnosol in rosemary and sage in the present study should be interpreted not only as a reflection of native plant chemistry, but also as a possible consequence of extraction-induced redistribution and oxidative conversion.

The detection of succinic acid in appreciable amounts in basil and sage, and across a broad range in rosemary, is noteworthy, although this compound is much less frequently highlighted in phytochemical reviews of *Lamiaceae* herbs than phenolic acids or diterpenes. Because the current literature places stronger chemotaxonomic emphasis on compounds such as rosmarinic acid, caffeic acid derivatives, flavonoids, and phenolic diterpenes, the distribution of succinic acid should be interpreted cautiously and primarily as a feature of the analyzed samples under the applied analytical conditions rather than as a universally recognized species-level marker. In contrast, the predominance of carnosol in rosemary and sage is strongly supported by the literature and fits the known diterpenoid antioxidant character of these herbs [21,49,50].

Overall, the present results reinforce the conclusion that oregano, basil, rosemary, and sage are rich in phenolic constituents, with rosmarinic acid emerging as a common major compound across all four of these herbs. At the same time, the stronger contribution of carnosol and related diterpenoid antioxidants in rosemary and sage differentiates these species from oregano and basil, which appear more clearly dominated by soluble phenolic acids and flavonoids. The simultaneous occurrence of high rosmarinic acid across all extracts and substantial quinic acid in selected matrices supports the view that these herbs are largely defined by hydroxycinnamate-related chemistry. Additionally, this demonstrates clear species-specific differences in secondary metabolite partitioning. Importantly, these differences should be interpreted as the combined result of intrinsic plant metabolism and extraction-driven selectivity and transformation, rather than as fixed compositional constants.

To enhance the scientific interpretation of these data, particular attention should be paid to the chemical markers that appear to drive differences in antioxidant activity among the samples. Rosmarinic acid, quinic acid, caffeoylquinic acid derivatives, and selected flavonoids provide useful indicators of the phytochemical character of individual herbs and mixtures. Their relative abundance reveals distinct metabolic signatures associated with botanical origin, formulation, and processing. For example, the predominance of hydroxycinnamate derivatives reflects a common *Lamiaceae*-type phenolic profile, whereas variation in caffeoylquinic acids and other minor constituents may distinguish specific commercial mixtures. These marker-based differences are relevant from a practical perspective because they may support the selection of herb materials with higher antioxidant potential for food preservation, functional food formulation, quality control of commercial spice mixtures, and the valorization of culinary herbs as natural sources of bioactive compounds.

### 3.3. PCA of Herbs

Principal Component Analysis (PCA) was conducted to examine the variability of 39 chemical compounds measured in various herb samples, including rosemary, sage, oregano, basil, herbs de Provence, Dalmatian herbs, and Sicilian herbs. PCA is a widely used statistical technique that reduces the dimensionality of complex datasets while retaining the most significant variance, facilitating the identification of underlying patterns and groupings among samples. The first three principal components (PC1, PC2, and PC3) collectively account for a significant portion of the total variance (62.14%), justifying their selection for visualization and interpretation. PC1 alone captures the largest proportion of variance (27.76%), indicating strong differences in the chemical composition of the examined herbs. PC2 and PC3 contribute additional variance, revealing finer distinctions among the samples (respectively, 17.85% and 16.53%).

**Figure 1:**
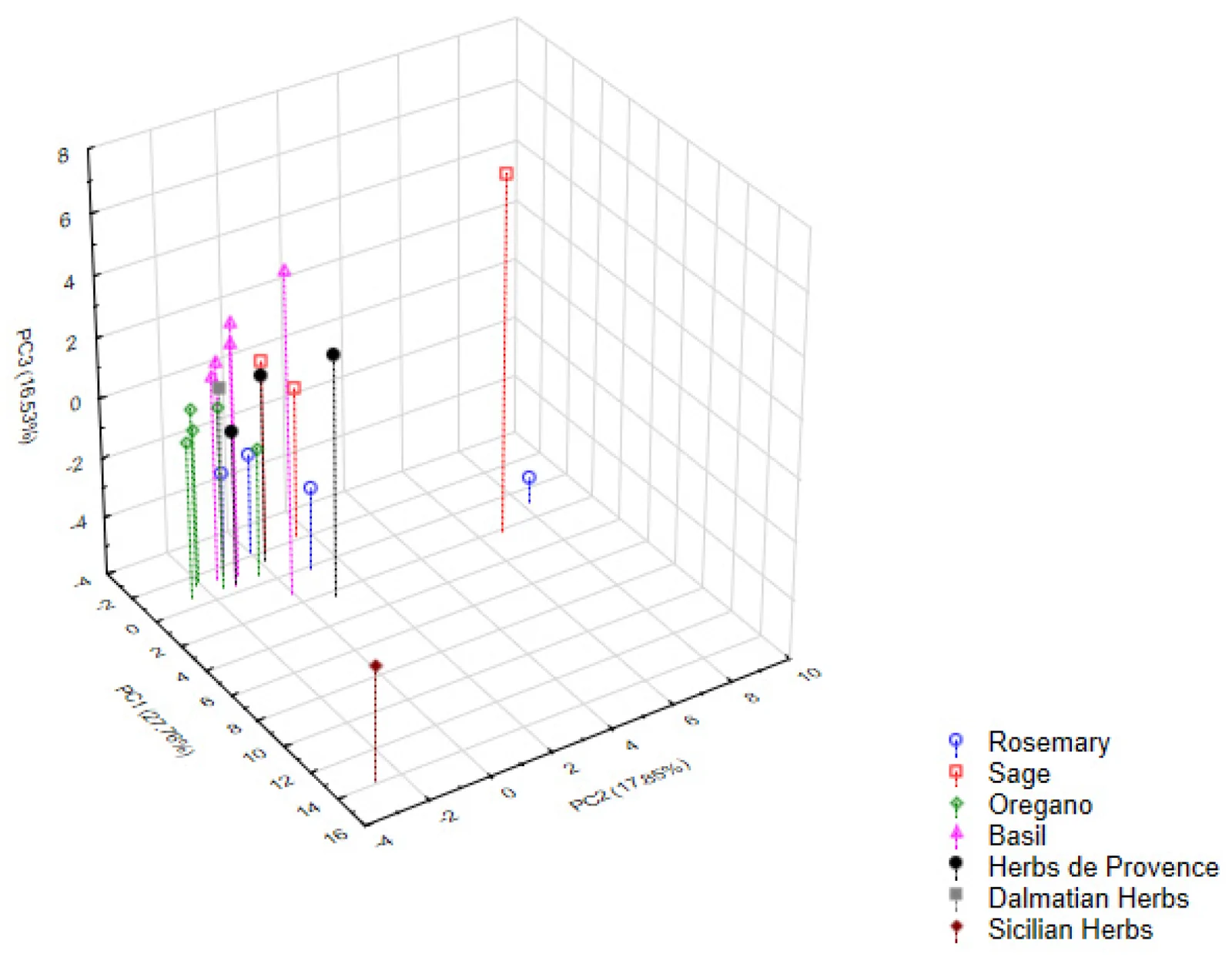
A three-dimensional PCA of herbs.

The three-dimensional scatter plot of PCA scores provides insight into the clustering patterns of herbal samples based on their chemical profiles (Figure 1). The samples are color-coded according to their respective herbal groups, allowing for a comparative assessment of intra-group and inter-group variability. The oregano and basil samples are positioned closely together in the PCA space when considering all principal components, showing some overlap. The shared clustering region of all these herbs may be attributed to common phytochemical constituents, such as flavonoids. Rosemary and sage are similar in terms of PC1 and PC3, suggesting great chemical similarity among these herbs. However, they display some separation in terms of PC2. Specifically, one sample from rosemary and one from sage exhibit significantly different chemical properties compared to other samples in their respective groups, resulting in distinct positioning in the PCA space. This deviation suggests potential variations in growing conditions, harvesting methods, or post-harvest processing, which may have influenced their chemical composition. The presence of such outliers underscores the complexity of herbal compositions and highlights the importance of detailed chemical profiling to understand the sources of variability within each herb type.

Herbs de Provence, Dalmatian herbs, and Sicilian herbs, being mixed herbal formulations, could display a broader distribution in PCA space, reflecting their composite nature. A greater dispersion of these samples was expected due to variability in their chemical composition, likely stemming from differences in herb proportions or variations in sourcing. However, herbs de Provence and Dalmatian herbs were identified among the single-herb samples. Only the Sicilian herbs exhibit the most pronounced deviation from the other groups, as evidenced by their distinct positioning in the PCA space—namely, a considerable variance along the PC1 axis. This reveals a unique chemical composition that sets Sicilian herbs apart from other formulations. And indeed, a roughly 10-fold higher concentration of 3-CQA was determined in the Sicilian herbs compared to other samples (Table 4). This elevated level may be attributed to the presence of dried tomatoes and tarragon, both of which are rich sources of 3-CQA [51,52]. Further targeted analysis of the specific compounds causing this differentiation could provide deeper insight into the chemical signatures of Sicilian herbs, as other factors may also shape their distinct profiles.

### 3.4. Spearman’s Correlation Analysis

Spearman’s correlation analysis was carried out to verify whether there was a relationship between the content of the main active substances and the results of the Folin–Ciocalteu test or the antioxidant activity measured in the ABTS and DPPH tests. Spearman’s correlation coefficient ranges from −1 to +1, and both −1 and +1 values indicate to a perfect correlation (−1 indicates a negative correlation—variable 2 decreases while variable 1 increases; +1 indicates a positive correlation—variable 2 increases while variable 1 increases). While there is no agreed or consistent interpretation for the obtained correlation meaning [53], an article by Schober et al. [54] reviews several approaches and presents ranges for different correlation levels from negligible to very strong (Table S23).

In the present study, Spearman’s correlation coefficient calculated for the concentration of rosmarinic acid and the Folin–Ciocalteu test results equals to 0.7030, indicating to a strong positive correlation between these two sets of results (Table S24). The *p*-value of 0.0003 proves that it is statistically significant. The relationship between rosmarinic acid concentration and ABTS assay results indicates to a moderate, statistically significant results (r = 0.6657, *p*-value = 0.0007—Table S25). The relationship between rosmarinic acid concentration and DPPH assay results is stronger, as r = 0.7188 and the *p*-value is 0.0002 (Table S26).

Similar calculations were also carried out for quinic acid, which is the second-most important compound detected in the samples tested, occurring in concentrations only slightly lower than those of rosmarinic acid. However, in this case, the correlation is not as high. The |r| values obtained (0.2027, 0.3529, and 0.1429 between quinic acid and Folin–Ciocalteu, ABTS, or DPPH results, respectively) indicate only a weak correlation between the results (Tables S27–S29). Also, the *p*-values are high (0.3656, 0.1072, and 0.5259, respectively). The results obtained clearly show that rosmarinic acid is the main compound responsible for the health-promoting properties of the herbs and herbal mixtures tested.

### 3.5. Research Limitations

Although extraction procedures are optimized to ensure the best possible yield of active substances, they do not guarantee the complete extraction of all compounds with antioxidant properties. The herbs under investigation may contain compounds with lower polarity that are not readily extracted with ethanol; however, their extraction might require the use of a more hazardous solvent.

The herbal mixtures under test were included for comparison with the four herbs that were the main focus of the study. However, the composition of these mixtures is known only in terms of their declared ingredients, but not in terms of their quantities.

## 4. Conclusions

The results of the present study have shown that herbs and their mixtures contain a large amount of various bioactive compounds and are a valuable source of them, so their consumption can bring many health benefits. The phytochemical profiles of oregano, basil, rosemary, and sage are consistent with the established chemistry of *Lamiaceae* herbs, particularly the widespread predominance of rosmarinic acid as a major non-volatile phenolic constituent. Furthermore, the study confirms that quantitative differences among species are anticipated and reflect the effects of genotype, developmental stage, environmental conditions, agronomic practices, and extraction methodology. The especially high rosmarinic acid content observed in oregano agrees with previous reports describing this herb as one of the richest culinary sources of phenolic antioxidants. Rosemary and sage displayed profiles that also included carnosol and related diterpenoid antioxidants, which are fully consistent with their known dual phytochemical character combining phenolic acids and lipophilic diterpenes. In basil, the profile characterized by high rosmarinic acid and relatively elevated rutin supports its classification as a herb rich in phenolic acids and flavonoids. The presence of quinic acids in oregano and rosemary is chemically plausible, although it should be regarded as an accessory component of phenolic metabolism rather than a primary chemotaxonomic marker. Overall, the results confirm that these herbs share a common hydroxycinnamate-based phytochemical framework while maintaining clear species-specific differences shaped by their intrinsic metabolism.

## Figures and Tables

**Table 1:** Abbreviations and names of compounds determined in herbs.

Abbreviation	Compound Name	Abbreviation	Compound Name
MEL	Melatonin	KAE	Kaempferol
TRG	Trigonelline	RUT	Rutin
NICAM	Nicotinamide	MYR	Myricetin
NICAC	Nicotinic acid	ROSM	Rosmarinic acid
THEA	Theanine	QUI	Quinic acid
SER	Serotonin	5-CQ	5-caffeoylquinic acid
THP	Theophylline	4-CQ	4-caffeoylquinic acid
CAF	Caffeine	3-CQ	3-caffeoylquinic acid
THB	Theobromine	3,4-DCQ	3,4-di-caffeoylquinic acid
FER	Ferulic acid	3,5-DCQ	3,5-di-caffeoylquinic acid
SYR	Syringic acid	4,5-DCQ	4,5-di-caffeoylquinic acid
GC	Gallocatechin	ECG	Epicatechin gallate
C	Catechin	CG	Catechin gallate
EC	Epicatechin	EGCG	Epigallocatechin gallate
CAR	Carnosol	GCG	Gallocatechin gallate
SIN	Synapic acid	GAL	Gallic acid
QUR	Quercetin	SUC	Succinic acid
SAL	Salicylic acid	PCCA	Protocatechuic acid
CAFAC	Caffeic acid	CARAC	Carnosic acid
COU	Coumaric acid		

**Table 2:** Herbs used in the experiments.

Sample Symbol	Brand	Ingredients
Pure herbs		
O1	Auchan	100% oregano
O2	Kotanyi	100% oregano
O3	Appetita	100% oregano
O4	Prymat	100% oregano
O5	Kamis	100% oregano
S1	Kotanyi	100% sage
S2	Vitax	100% sage
S3	Belin	100% sage
B1	Auchan	100% basil
B2	Kotanyi	100% basil
B3	Kamis	100% basil
B4	Appetita	100% basil
B5	Prymat	100% basil
R1	Herbes	100% rosemary
R2	Kamis	100% rosemary
R3	Kotanyi	100% rosemary
R4	Bystry	100% rosemary
Herbal mixtures		
HP1	Kotanyi	Herbes de Provence: rosemary, thyme, savory, basil, tarragon
HP2	Kamis	Herbes de Provence: basil, marjoram, rosemary, savory, sage, oregano, thyme, mint
HP3	Prymat	Herbes de Provence: thyme, basil, savory, marjoram, rosemary, oregano, bay leaves
DH1	Kotanyi	Dalmatian herbs: oregano, basil, marjoram, rosemary, savory, thyme, sage
SH1	Kamis	Sicilian herbs: dried tomatoes, basil, parsley, onion, oregano, garlic, thyme, savory, tarragon

**Table 3:** Test results obtained for all herbs and herbal mixtures (on heat maps, high values are shown in red, followed by progressively lower values–dark orange, light orange, light green and dark green; each column treated separately).

Samples	Folin–Ciocalteu [mg GAE/mL]	ABTS [mg Trolox/mL]	DPPH [mg Trolox/mL]
Oregano			
O1	2.3873 ± 0.0289	3.9097 ± 0.0741	2.4029 ± 0.0092
O2	2.4453 ± 0.0450	4.3126 ± 0.0391	2.6867 ± 0.0787
O3	2.2868 ± 0.0053	3.9937 ± 0.1198	2.3159 ± 0.0383
O4	3.7304 ± 0.1255	6.1835 ± 0.2062	6.7007 ± 0.1464
O5	2.2598 ± 0.0661	4.3646 ± 0.0294	2.3672 ± 0.0569
Sage			
S1	2.4463 ± 0.0501	3.4981 ± 0.1157	3.3675 ± 0.0203
S2	2.3148 ± 0.0052	3.5494 ± 0.1338	3.4000 ± 0.0264
S3	0.6708 ± 0.0005	1.1762 ± 0.0156	0.9356 ± 0.0263
Basil			
B1	1.6623 ± 0.0118	2.2183 ± 0.0501	2.2694 ± 0.0290
B2	1.5505 ± 0.0483	2.1733 ± 0.0523	2.1245 ± 0.0064
B3	2.2568 ± 0.0022	3.5415 ± 0.0651	3.3170 ± 0.0600
B4	2.2191 ± 0.0778	3.5893 ± 0.0219	3.1457 ± 0.0367
B5	2.0364 ± 0.0236	4.0403 ± 0.0705	3.2891 ± 0.0219
Rosemary			
R1	1.1033 ± 0.0187	1.4131 ± 0.0486	1.3831 ± 0.0237
R2	1.1141 ± 0.0156	1.3531 ± 0.0399	1.3214 ± 0.0397
R3	1.3710 ± 0.0032	1.6578 ± 0.0429	1.2465 ± 0.0096
R4	1.1057 ± 0.0317	1.6452 ± 0.0465	1.2362 ± 0.0405
Herbes de Provence			
HP1	1.6035 ± 0.0366	2.2452 ± 0.0585	2.0065 ± 0.0405
HP2	2.8317 ± 0.0126	4.0635 ± 0.0961	3.3628 ± 0.0033
HP3	2.5189 ± 0.0228	4.5919 ± 0.0739	2.6072 ± 0.0170
Dalmatian herbs			
DH1	1.9874 ± 0.0613	2.9268 ± 0.0529	2.1926 ± 0.0250
Sicilian herbs			
SH1	1.6575 ± 0.0354	2.8712 ± 0.0391	1.5100 ± 0.0409

**Table 4:** Content of bioactive compounds in the herbs, µg/g ± standard deviation (on heat maps, high values are shown in red, followed by progressively lower values–dark orange, light orange, light green and dark green; each column treated separately).

**Sample**	**MEL**	**TRG**	**NICAM**	**NICAC**	**THEA**	**SER**	**THP**	**CAF**	**SIN**	**THB**	**FER**	**SYR**	**GC**	**C**	**EC**
O1	<LOQ	3.78 ± 0.13	4.11 ± 0.15	2.47 ± 0.07	0.61 ± 0.01	0.32 ± 0.004	<LOD	0.01 ± 0.0004	<LOQ	<LOQ	5.67 ± 0.17	14.17 ± 0.29	0.99 ± 0.02	1.24 ± 0.05	7.77 ± 0.27
O2	<LOD	3.71 ± 0.09	5.36 ± 0.16	2.09 ± 0.06	0.52 ± 0.01	0.49 ± 0.02	<LOD	0.01 ± 0.0003	0.47 ± 0.02	<LOD	7.45 ± 0.19	13.21 ± 0.54	<LOD	0.26 ± 0.005	0.94 ± 0.03
O3	<LOQ	2.89 ± 0.09	4.89 ± 0.15	2.07 ± 0.03	0.44 ± 0.01	<LOQ	<LOD	0.01 ± 0.0004	<LOQ	<LOQ	5.71 ± 0.15	16.58 ± 0.64	3.47 ± 0.11	1.47 ± 0.04	0.42 ± 0.01
O4	<LOQ	7.92 ± 0.32	4.03 ± 0.07	1.59 ± 0.03	0.42 ± 0.008	<LOQ	<LOD	0.01 ± 0.0002	<LOQ	<LOQ	18.02 ± 0.71	3.41 ± 0.08	0.37 ± 0.01	1.91 ± 0.07	0.99 ± 0.02
O5	<LOD	0.78 ± 0.01	4.32 ± 0.15	1.48 ± 0.05	0.49 ± 0.02	0.41 ± 0.01	<LOD	0.01 ± 0.0006	<LOQ	<LOD	6.03 ± 0.17	8.76 ± 0.36	0.27 ± 0.006	<LOQ	<LOQ
S1	<LOD	0.15 ± 0.003	2.12 ± 0.06	0.94 ± 0.03	0.21 ± 0.005	<LOD	<LOQ	0.005 ± 0.0003	<LOQ	<LOQ	10.98 ± 0.39	3.47 ± 0.08	<LOD	0.19 ± 0.004	0.56 ± 0.01
S2	<LOD	0.88 ± 0.02	5.12 ± 0.16	2.47 ± 0.11	1.11 ± 0.04	<LOQ	<LOQ	0.04 ± 0.001	2.49 ± 0.10	0.15 ± 0.003	38.54 ± 1.16	15.18 ± 0.36	<LOQ	4.86 ± 0.18	0.43 ± 0.006
S3	<LOD	0.75 ± 0.02	0.98 ± 0.04	2.45 ± 0.07	0.65 ± 0.02	<LOQ	<LOQ	0.01 ± 0.001	1.31 ± 0.04	<LOQ	9.24 ± 0.23	4.28 ± 0.06	<LOQ	0.47 ± 0.007	0.33 ± 0.006
B1	<LOD	6.47 ± 0.14	5.99 ± 0.25	1.87 ± 0.04	1.24 ± 0.02	0.32 ± 0.008	<LOD	0.01 ± 0.0004	<LOQ	<LOD	5.13 ± 0.17	3.44 ± 0.11	<LOD	0.95 ± 0.04	0.21 ± 0.003
B2	<LOD	3.13 ± 0.08	6.42 ± 0.09	1.59 ± 0.05	0.99 ± 0.03	0.29 ± 0.01	<LOD	0.01 ± 0.0003	<LOQ	<LOD	4.47 ± 0.07	2.54 ± 0.10	<LOD	1.48 ± 0.06	<LOQ
B3	<LOD	0.85 ± 0.02	4.85 ± 0.17	1.46 ± 0.02	0.95 ± 0.02	0.25 ± 0.004	<LOD	0.01 ± 0.0004	<LOQ	<LOQ	5.09 ± 0.16	2.75 ± 0.07	<LOD	0.19 ± 0.003	<LOD
B4	<LOD	0.98 ± 0.03	5.86 ± 0.23	1.64 ± 0.05	0.97 ± 0.04	0.37 ± 0.01	<LOD	0.01 ± 0.0003	<LOQ	<LOD	5.37 ± 0.14	3.43 ± 0.11	<LOD	0.38 ± 0.008	<LOD
B5	<LOQ	7.26 ± 0.31	7.98 ± 0.23	2.85 ± 0.12	0.89 ± 0.02	0.29 ± 0.01	<LOQ	0.02 ± 0.0002	0.89 ± 0.04	<LOQ	7.42 ± 0.27	3.40 ± 0.09	<LOQ	0.58 ± 0.01	<LOQ
R1	<LOD	<LOQ	0.58 ± 0.02	0.16 ± 0.006	0.12 ± 0.004	<LOD	<LOQ	0.01 ± 0.0004	<LOQ	<LOQ	9.09 ± 0.20	3.85 ± 0.05	<LOD	<LOD	<LOD
R2	<LOD	<LOQ	0.98 ± 0.02	0.17 ± 0.003	0.08 ± 0.002	<LOD	<LOQ	0.01 ± 0.0005	<LOQ	<LOQ	9.84 ± 0.39	4.91 ± 0.07	<LOD	<LOD	<LOD
R3	<LOD	<LOQ	1.91 ± 0.05	0.25 ± 0.007	0.10 ± 0.003	<LOQ	<LOQ	0.02 ± 0.001	2.05 ± 0.08	<LOQ	17.82 ± 0.33	7.57 ± 0.23	<LOQ	<LOQ	<LOQ
R4	<LOD	0.13 ± 0.002	0.83 ± 0.02	0.27 ± 0.007	0.18 ± 0.004	<LOQ	0.06 ± 0.002	0.02 ± 0.0003	0.89 ± 0.03	<LOQ	10.23 ± 0.39	3.01 ± 0.07	<LOQ	0.62 ± 0.02	<LOQ
HP1	<LOD	3.21 ± 0.06	4.08 ± 0.12	1.21 ± 0.02	0.51 ± 0.02	0.37 ± 0.01	<LOD	0.01 ± 0.0004	<LOQ	<LOD	6.97 ± 0.13	22.89 ± 0.41	<LOD	1.71 ± 0.07	<LOQ
HP2	<LOD	0.67 ± 0.02	4.59 ± 0.09	1.34 ± 0.02	0.78 ± 0.02	0.22 ± 0.003	<LOQ	0.01 ± 0.0003	<LOQ	<LOD	11.06 ± 0.43	10.57 ± 0.19	1.35 ± 0.02	0.89 ± 0.02	<LOD
HP3	<LOD	3.75 ± 0.09	7.87 ± 0.24	3.96 ± 0.09	0.79 ± 0.02	0.30 ± 0.005	<LOQ	0.03 ± 0.001	1.03 ± 0.03	0.11 ± 0.003	9.88 ± 0.32	12.50 ± 0.26	<LOQ	0.45 ± 0.01	6.26 ± 0.25
DH1	<LOD	0.89 ± 0.03	5.13 ± 0.11	1.65 ± 0.05	0.75 ± 0.03	0.27 ± 0.008	<LOD	0.007 ± 0.0002	<LOQ	<LOD	6.05 ± 0.21	11.54 ± 0.36	0.50 ± 0.01	0.22 ± 0.003	0.48 ± 0.01
SH1	<LOD	164.12 ± 6.94	21.84 ± 0.77	1.74 ± 0.03	1.07 ± 0.02	0.41 ± 0.01	0.06 ± 0.002	0.03 ± 0.001	0.95 ± 0.01	0.12 ± 0.004	8.24 ± 0.33	6.97 ± 0.13	<LOQ	0.17 ± 0.003	<LOQ
**Sample**	**CAR**	**SIN**	**QUR**	**SAL**	**CAFAC**	**COU**	**KAE**	**RUT**	**MYR**	**ROSM**	**QUI**	**5-CQ**	**4-CQ**		
O1	0.17 ± 0.005	<LOQ	3.13 ± 0.09	12.69 ± 0.52	45.55 ± 1.10	4.24 ± 0.14	6.49 ± 0.17	87.57 ± 1.94	<LOD	3645.41 ± 87.69	7884.51 ± 333.14	24.31 ± 1.03	23.02 ± 0.71		
O2	0.18 ± 0.0045	0.11 ± 0.002	2.57 ± 0.11	9.38 ± 0.29	86.98 ± 2.75	3.06 ± 0.06	6.11 ± 0.23	13.09 ± 0.52	<LOD	7861.25 ± 237.98	6264.72 ± 151.44	9.71 ± 0.16	12.51 ± 0.54		
O3	0.24 ± 0.0047	0.14 ± 0.003	4.57 ± 0.19	13.39 ± 0.59	91.06 ± 2.65	3.46 ± 0.06	8.24 ± 0.21	7.61 ± 0.23	<LOQ	5962.49 ± 96.41	7136.01 ± 198.65	7.98 ± 0.18	13.27 ± 0.22		
O4	0.06 ± 0.0015	0.21 ± 0.01	2.52 ± 0.04	3.30 ± 0.05	25.51 ± 0.40	2.07 ± 0.07	10.02 ± 0.21	45.78 ± 1.58	<LOD	9800.51 ± 321.36	4078.11 ± 176.69	18.15 ± 0.31	24.01 ± 0.53		
O5	0.03 ± 0.0008	<LOQ	1.79 ± 0.04	7.22 ± 0.26	29.02 ± 1.13	1.89 ± 0.04	4.34 ± 0.08	5.70 ± 0.21	<LOD	5771.20 ± 188.65	6357.87 ± 174.14	5.91 ± 0.15	10.32 ± 0.37		
S1	72.14 ± 1.59	0.56 ± 0.01	1.52 ± 0.05	15.91 ± 0.62	112.30 ± 2.89	3.24 ± 0.05	3.41 ± 0.07	7.11 ± 0.25	<LOD	7721.50 ± 293.85	19.21 ± 0.56	4.57 ± 0.08	<LOD		
S2	38.67 ± 1.67	2.47 ± 0.09	0.41 ± 0.02	82.17 ± 1.35	258.54 ± 8.12	5.31 ± 0.21	3.62 ± 0.12	22.99 ± 0.58	<LOQ	15,556.21 ± 690.92	24.87 ± 0.78	5.57 ± 0.09	2.23 ± 0.05		
S3	28.24 ± 1.01	0.67 ± 0.01	0.47 ± 0.01	11.45 ± 0.34	133.71 ± 4.41	2.04 ± 0.06	0.99 ± 0.03	9.65 ± 0.25	<LOQ	1041.40 ± 16.06	65.23 ± 2.39	2.19 ± 0.08	1.22 ± 0.04		
B1	0.01 ± 0.0003	0.31 ± 0.01	0.15 ± 0.002	79.71 ± 2.16	155.14 ± 3.48	3.31 ± 0.07	<LOQ	352.15 ± 9.19	<LOD	6731.05 ± 121.64	1450.79 ± 30.28	10.25 ± 0.29	8.62 ± 0.19		
B2	0.02 ± 0.0007	0.24 ± 0.004	0.08 ± 0.002	18.08 ± 0.33	92.10 ± 1.50	3.31 ± 0.07	0.35 ± 0.01	369.13 ± 11.24	<LOD	3510.00 ± 78.10	1603.04 ± 24.00	2.07 ± 0.04	2.48 ± 0.04		
B3	0.01 ± 0.0003	0.18 ± 0.004	0.06 ± 0.003	16.31 ± 0.26	97.93 ± 3.84	3.07 ± 0.05	<LOQ	779.41 ± 27.27	<LOD	6540.10 ± 101.35	1629.17 ± 64.90	1.27 ± 0.03	1.08 ± 0.03		
B4	0.01 ± 0.0002	0.25 ± 0.01	0.13 ± 0.003	64.81 ± 2.46	140.74 ± 4.25	3.95 ± 0.10	<LOQ	849.35 ± 20.21	<LOD	8157.89 ± 239.84	1573.33 ± 52.55	0.65 ± 0.01	0.71 ± 0.03		
B5	0.05 ± 0.0012	0.36 ± 0.01	0.07 ± 0.002	115.17 ± 3.13	273.03 ± 7.85	6.48 ± 0.15	0.20 ± 0.01	1229.99 ± 43.09	<LOQ	8181.26 ± 264.97	877.06 ± 29.59	18.48 ± 0.39	19.51 ± 0.72		
R1	22.91 ± 0.57	0.40 ± 0.01	0.81 ± 0.03	2.78 ± 0.06	35.09 ± 1.15	3.72 ± 0.16	0.47 ± 0.02	8.82 ± 0.15	<LOD	1121.15 ± 39.63	3087.51 ± 49.66	10.21 ± 0.24	29.14 ± 1.07		
R2	28.79 ± 1.20	0.57 ± 0.02	0.66 ± 0.01	3.19 ± 0.05	20.35 ± 0.52	7.22 ± 0.17	0.54 ± 0.01	6.58 ± 0.10	<LOD	2622.34 ± 109.94	1405.54 ± 37.82	9.80 ± 0.36	27.85 ± 1.04		
R3	109.57 ± 1.80	0.92 ± 0.05	1.08 ± 0.04	4.28 ± 0.09	48.41 ± 1.07	16.86 ± 0.38	12.03 ± 0.31	9.91 ± 0.40	0.25 ± 0.01	9581.24 ± 399.16	6149.56 ± 160.46	30.04 ± 0.64	61.31 ± 2.02		
R4	49.15 ± 1.82	0.49 ± 0.02	0.77 ± 0.03	4.46 ± 0.10	42.94 ± 1.33	2.98 ± 0.12	12.14 ± 0.54	14.26 ± 0.34	<LOQ	1504.05 ± 45.27	5973.33 ± 183.98	17.43 ± 0.67	41.54 ± 1.57		
HP1	3.59 ± 0.12	0.45 ± 0.01	2.76 ± 0.12	8.78 ± 0.33	85.14 ± 1.31	3.26 ± 0.07	2.54 ± 0.05	207.19 ± 8.86	<LOD	4624.78 ± 115.85	1481.16 ± 33.35	15.38 ± 0.53	32.18 ± 1.14		
HP2	9.12 ± 0.33	0.47 ± 0.01	1.51 ± 0.04	11.23 ± 0.26	120.01 ± 2.15	3.51 ± 0.13	3.44 ± 0.11	201.14 ± 5.21	<LOQ	9333.71 ± 287.73	3710.07 ± 97.11	10.87 ± 0.13	19.99 ± 0.37		
HP3	3.55 ± 0.14	0.51 ± 0.01	5.13 ± 0.20	15.13 ± 0.57	180.08 ± 6.03	5.25 ± 0.22	12.57 ± 0.40	497.90 ± 14.81	<LOQ	10,254.61 ± 437.04	5239.51 ± 153.66	12.51 ± 0.47	33.79 ± 1.27		
DH1	8.27 ± 0.29	0.32 ± 0.01	2.71 ± 0.11	12.08 ± 0.35	89.75 ± 3.47	3.58 ± 0.07	3.94 ± 0.11	85.94 ± 3.21	<LOD	7684.16 ± 338.34	3395.17 ± 139.53	6.80 ± 0.13	11.98 ± 0.32		
SH1	0.10 ± 0.001	0.39 ± 0.01	35.15 ± 1.57	10.81 ± 0.36	89.32 ± 2.47	4.56 ± 0.09	13.82 ± 0.57	810.54 ± 22.53	<LOQ	5491.23 ± 229.86	7307.08 ± 272.94	96.95 ± 2.92	76.86 ± 1.71		
**Sample**	**3-CQ**	**3.4-DCQ**	**3.5-DCQ**	**4.5-DCQ**	**ECG**	**CG**	**EGCG**	**GCG**	**GAL**	**SUC**	**PCCA**	**CARAC**			
O1	139.09 ± 3.93	2.16 ± 0.06	9.45 ± 0.17	3.83 ± 0.05	0.24 ± 0.01	0.22 ± 0.01	<LOQ	<LOD	1.84 ± 0.04	67.46 ± 1.14	71.41 ± 2.02	2.22 ± 0.01			
O2	58.78 ± 2.06	2.54 ± 0.03	10.99 ± 0.41	4.57 ± 0.17	<LOD	<LOQ	<LOD	<LOD	1.32 ± 0.03	71.07 ± 1.91	49.31 ± 1.51	2.91 ± 0.10			
O3	53.51 ± 2.39	1.43 ± 0.01	4.73 ± 0.10	2.93 ± 0.08	<LOD	<LOQ	<LOQ	<LOD	3.25 ± 0.19	66.54 ± 1.50	53.28 ± 1.78	7.01 ± 0.22			
O4	71.25 ± 2.60	9.84 ± 0.26	25.67 ± 1.20	12.49 ± 0.37	0.59 ± 0.01	0.31 ± 0.01	<LOD	<LOD	1.78 ± 0.04	72.51 ± 2.39	35.99 ± 0.10	<LOQ			
O5	35.82 ± 0.67	0.92 ± 0.01	3.04 ± 0.03	2.49 ± 0.01	0.14 ± 0.01	<LOQ	<LOD	<LOD	1.48 ± 0.04	54.22 ± 0.85	32.88 ± 1.04	<LOQ			
S1	14.51 ± 0.27	1.01 ± 0.04	10.01 ± 0.19	4.16 ± 0.09	<LOQ	<LOQ	<LOD	<LOD	0.95 ± 0.02	323.74 ± 5.44	11.26 ± 0.20	441.23 ± 12.08			
S2	17.74 ± 0.55	0.49 ± 0.01	2.87 ± 0.05	0.67 ± 0.01	0.21 ± 0.01	<LOQ	0.91 ± 0.03	<LOQ	2.02 ± 0.07	688.16 ± 29.80	15.65 ± 0.38	159.44 ± 4.08			
S3	16.10 ± 0.52	<LOQ	2.51 ± 0.04	0.48 ± 0.01	0.26 ± 0.01	<LOQ	<LOQ	<LOQ	0.99 ± 0.02	288.40 ± 7.29	18.20 ± 0.66	55.20 ± 0.74			
B1	95.06 ± 1.67	6.10 ± 0.19	34.96 ± 0.70	9.85 ± 0.29	0.71 ± 0.02	<LOD	<LOD	<LOD	2.32 ± 0.03	404.86 ± 17.85	19.92 ± 0.31	<LOD			
B2	30.45 ± 0.87	1.66 ± 0.03	7.08 ± 0.22	2.65 ± 0.05	0.45 ± 0.01	0.28 ± 0.01	<LOD	<LOD	2.59 ± 0.06	402.86 ± 15.59	16.83 ± 0.39	<LOD			
B3	13.51 ± 0.27	1.56 ± 0.07	3.85 ± 0.08	1.61 ± 0.03	<LOQ	<LOQ	<LOD	<LOD	1.60 ± 0.05	397.14 ± 13.70	19.27 ± 0.43	<LOD			
B4	10.31 ± 0.14	<LOQ	2.99 ± 0.01	1.41 ± 0.01	<LOD	<LOD	<LOD	<LOD	2.29 ± 0.06	645.71 ± 27.64	18.78 ± 0.59	<LOD			
B5	148.09 ± 4.33	11.48 ± 0.47	27.41 ± 1.26	15.01 ± 0.53	<LOD	<LOD	<LOD	<LOD	1.59 ± 0.05	847.40 ± 34.40	39.02 ± 1.56	0.41 ± 0.01			
R1	2.66 ± 0.09	0.81 ± 0.03	0.73 ± 0.03	0.35 ± 0.01	0.41 ± 0.01	0.48 ± 0.01	<LOD	<LOD	<LOD	68.14 ± 1.22	5.15 ± 0.11	14.50 ± 0.03			
R2	1.95 ± 0.04	<LOD	<LOQ	<LOD	0.95 ± 0.02	0.11 ± 0.003	<LOD	<LOD	<LOQ	86.57 ± 3.19	6.61 ± 0.24	23.38 ± 1.05			
R3	6.43 ± 0.20	3.58 ± 0.13	4.80 ± 0.14	3.87 ± 0.12	<LOQ	0.19 ± 0.01	0.71 ± 0.02	0.26 ± 0.03	<LOQ	461.41 ± 19.51	11.75 ± 0.47	546.17 ± 13.68			
R4	4.72 ± 0.12	<LOQ	0.97 ± 0.03	0.48 ± 0.01	0.16 ± 0.003	0.13 ± 0.004	0.69 ± 0.02	<LOQ	1.30 ± 0.05	431.43 ± 10.48	10.00 ± 0.35	94.51 ± 2.30			
HP1	141.43 ± 2.51	5.48 ± 0.09	49.14 ± 0.94	41.20 ± 1.12	0.48 ± 0.01	0.30 ± 0.01	<LOD	<LOD	1.18 ± 0.04	86.25 ± 3.24	7.51 ± 0.29	59.85 ± 2.21			
HP2	12.31 ± 0.32	0.35 ± 0.01	1.36 ± 0.06	1.91 ± 0.03	<LOQ	<LOQ	<LOD	<LOD	2.64 ± 0.11	327.97 ± 12.65	14.80 ± 0.45	520.59 ± 10.46			
HP3	42.8 ± 1.22	1.77 ± 0.03	7.87 ± 0.13	4.45 ± 0.07	0.48 ± 0.02	<LOQ	0.66 ± 0.02	<LOD	2.01 ± 0.05	838.01 ± 29.15	22.60 ± 0.50	19.56 ± 0.54			
DH1	22.22 ± 0.79	0.70 ± 0.03	3.47 ± 0.07	1.75 ± 0.04	<LOD	<LOQ	<LOD	<LOD	2.57 ± 0.08	235.16 ± 10.52	27.72 ± 1.18	6.59 ± 0.26			
SH1	1021.21 ± 23.24	32.69 ± 1.45	660.31 ± 17.65	227.80 ± 3.60	0.46 ± 0.02	<LOQ	1.98 ± 0.07	<LOD	1.99 ± 0.03	521.41 ± 14.93	20.41 ± 0.91	0.71 ± 0.02			

## Data Availability

Data is contained within the article or Supplementary Materials.

## Supplementary Materials

The following supporting information can be downloaded at https://www.mdpi.com/article/10.3390/antiox15091113/s1, Table S1. Experimental parameters used in herb extraction optimization; Table S2. Mass spectrometer parameters applied for the determination of compounds; Table S3. LC-MS/MS parameters for determined compounds (RT—retention time; LOD—limit of detection; LOQ—limit of quantification); Table S4. Oregano extraction optimization test results; Table S5. Sage extraction optimization test results; Table S6. Basil extraction optimization test results; Table S7. Rosemary extraction optimization test results; Table S8. The response surfaces in the tests Folin–Ciocalteu, ABTS, and DPPH for herbs; Table S9. Pareto charts in the tests Folin–Ciocalteu, ABTS, and DPPH for herbs; Table S10. Optimization of oregano extraction towards the greatest total phenolic content determined by the Folin–Ciocalteu test (R^2^: 0.9263, Adjusted R^2^: 0.8736, Predicted R^2^: 0.6940, C.V. %: 13.83, Adequate Precision: 11.384; y = 0.034422 + (0.075491)·A + (0.049007)·B + (−0.001685)·A^2^ + (−0.000545)·B^2^ + (0.000064)·AB); Table S11. Optimization of oregano extraction towards the greatest antioxidant activity determined by the ABTS assay (R^2^: 0.9323, Adjusted R^2^: 0.8840, Predicted R^2^: 0.7375, C.V. %: 11.41, Adequate Precision: 12.318; y = 0.437440 + (0.044429)·A + (0.066649)·B + (−0.001132)·A^2^ + (−0.000711)·B^2^ + (0.000452)·AB); Table S12. Optimization of oregano extraction towards the greatest antioxidant activity determined by the DPPH assay (R^2^: 0.8370, Adjusted R^2^: 0.7206, Predicted R^2^: 0.2725, C.V. %: 25.70, Adequate Precision: 9.499; y = 0.825632 + (−0.036325)·A + (0.061500)·B + (0.002279)·A^2^ + (−0.000631)·B^2^ + (−0.000457)·AB); Table S13. Optimization of sage extraction towards the greatest total phenolic content determined by the Folin–Ciocalteu test (R^2^: 0.9701, Adjusted R^2^: 0.9487, Predicted R^2^: 0.9120, C.V. %: 10.38, Adequate Precision: 21.503; y = −0.667450 + (0.053001)·A + (0.050244)·B + (−0.000454)·A^2^ + (−0.000435)·B^2^ + (−0.000091)·AB); Table S14. Optimization of sage extraction towards the greatest antioxidant activity determined by the ABTS assay (R^2^: 0.9869, Adjusted R^2^: 0.9776, Predicted R^2^: 0.9434, C.V. %: 6.86, Adequate Precision: 30.508; y = −1.243599 + (0.112881)·A + (0.072589)·B + (−0.001663)·A^2^ + (−0.000630)·B^2^ + (−0.000164)·AB); Table S15. Optimization of sage extraction towards the greatest antioxidant activity determined by the DPPH assay (R^2^: 0.8609, Adjusted R^2^: 0.7616, Predicted R^2^: 0.1229, C.V. %: 21.21, Adequate Precision: 9.548; y = −0.785222 + (0.035326)·A + (0.088152)·B + (0.000650)·A^2^ + (−0.000569)·B^2^ + (−0.000750)·AB); Table S16. Optimization of basil extraction towards the greatest total phenolic content determined by the Folin–Ciocalteu test (R^2^: 0.9337, Adjusted R^2^: 0.8864, Predicted R^2^: 0.6493, C.V. %: 14.99, Adequate Precision: 14.765; y = 1.340451 + (0.000218)·A + (0.004651)·B + (0.000451)·A^2^ + (−0.000187)·B^2^ + (−0.000031)·AB); Table S17. Optimization of basil extraction towards the greatest antioxidant activity determined by the ABTS assay (R^2^: 0.9371, Adjusted R^2^: 0.8922, Predicted R^2^: 0.6659, C.V. %: 15.15, Adequate Precision: 15.308; y = 1.333213 + (0.033791)·A + (0.012594)·B + (0.000044)·A^2^ + (−0.000289)·B^2^ + (−0.000177)·AB); Table S18. Optimization of basil extraction towards the greatest antioxidant activity determined by the DPPH assay (R^2^: 0.8708, Adjusted R^2^: 0.7786, Predicted R^2^: 0.3691, C.V. %: 23.62, Adequate Precision: 10.778; y = 1.806362 + (−0.011892)·A + (0.015333)·B + (0.001814)·A^2^ + (−0.000323)·B^2^ + (−0.000320)·AB); Table S19. Optimization of rosemary extraction towards the greatest total phenolic content determined by the Folin–Ciocalteu test (R^2^: 0.7477; Adjusted R^2^: 0.5675; Predicted R^2^: 0.0854; C.V. %: 20.20; Adequate Precision: 5.822; y = 0.161047 + (0.015350)·A + (0.007546)·B + (−0.000325)·A^2^ + (−0.000101)·B^2^ + (0.000157)·AB); Table S20. Optimization of rosemary extraction towards the greatest antioxidant activity determined by the ABTS assay (R^2^: 0.7990, Adjusted R^2^: 0.6554, Predicted R^2^: 0.2746, C.V. %: 20.70, Adequate Precision: 6.608; y = 0.056221 + (0.027282)·A + (0.014853)·B + (−0.000467)·A^2^ + (−0.000170)·B^2^ + (0.000076)·AB); Table S21. Optimization of rosemary extraction towards the greatest antioxidant activity determined by the DPPH assay (R^2^: 0.8224, Adjusted R^2^: 0.6955, Predicted R^2^: 0.5685, C.V. %: 18.52, Adequate Precision: 6.865; y = −0.041776 + (0.037881)·A + (0.016518)·B + (−0.000551)·A^2^ + (−0.000171)·B^2^ + (0.000072)·AB); Table S22. Extraction parameters obtained for herbs; Table S23. Spearman’s correlation coefficients meaning; Table S24. Spearman’s correlation between the content of rosmarinic acid (ROSM) and the total phenolic content (the Folin–Ciocalteu test results); Table S25. Spearman’s correlation between the content of rosmarinic acid (ROSM) and the antioxidant activity (ABTS assay results); Table S26. Spearman’s correlation between the content of rosmarinic acid (ROSM) and the antioxidant activity (DPPH assay results); Table S27. Spearman’s correlation between the content of quinic acid (QUI) and the total phenolic content (the Folin–Ciocalteu test results); Table S28. Spearman’s correlation between the content of quinic acid (QUI) and the antioxidant activity (ABTS assay results); Table S29. Spearman’s correlation between the content of quinic acid (QUI) and the antioxidant activity (DPPH assay results); Figure S1. Chromatograms of (a) standard solution, (b) sample 10, (c) sample 8.
