# Supplementary material for: Antioxidant Properties and Bioactive Compounds of Oregano, Sage, Basil, Rosemary, and Herbal Mixtures
Source: Antioxidants (Basel). 2026 Sep 4;15(9):1113. doi: 10.3390/antiox15091113 (PMC13603443; doi:10.3390/antiox15091113)
Supplement: Supplementary file 1 [file antioxidants-15-01113-s001.zip › antioxidants-4498104-supplementary.pdf]

Supplement

Antioxidant properties and bioactive compounds of oregano, sage, basil, rosemary, and herbal mixtures

Julia Płatkiewicz[1], Joanna, Wróbel[1], Magdalena Jeszka-Skowron[1], Robert Frankowski[1],

Zuzanna Grześkowiak[2], Beata Czarczyńska-Goślińska[2], Anna Maria Jeszka[3],

Agnieszka Zgoła-Grześkowiak[1,*]

[1] Institute of Chemistry and Technical Electrochemistry, Poznan University of Technology, Berdychowo 4, 60-965 Poznań, Poland

[2] Department of Pharmaceutical Technology, Poznan University of Medical Sciences, Grunwaldzka 6, 60-780 Poznań, Poland

[3] Institute of Socio-Economics, Poznan University of Economics and Business, Al. Niepodległości 10, 61-875 Poznań, Poland

Table S1. Experimental parameters used in herb extraction optimization

Experiment	Parameters	
	Time [min]	Ethanol content [%]
1	8.7	14.6
2	8.7	85.4
3	26.3	14.6
4	26.3	85.4
5	5	50
6	30	50
7	17.5	0
8	17.5	100
9	17.5	50
10	17.5	50
11	17.5	50
12	17.5	50
13	17.5	50

Table S2. Mass spectrometer parameters applied for the determination of compounds

Compound	Abbr.	DP[a] [V]	Analytical transition	CE[b] [eV]	EP[c] [V]	CXP[d] [V]	Confirmatory transition	CE [eV]	EP [V]	CXP [V]
**Positive ionization**										
Melatonin	MEL	85	233 → 174	19	5	10	233 → 159	40	6	10
Trigonelline	TRG	50	138 → 94	30	10	4	138 → 110	30	10	5
Nicotinamide	NICAM	20	123 → 80	28	10	2	123 → 96	28	10	4
Nicotinic acid	NICAC	30	124 → 80	30	10	3	124 → 78	32	10	3
Theanine	THEA	35	175 → 84	30	10	4	175 → 158	17	10	9
Serotonin	SER	50	177 → 160	9	10	12	177 → 132	31	6	7
Theophylline	THP	50	181 → 124	27	10	6	181 → 96	33	10	4
Caffeine	CAF	25	195 → 138	26	10	5	195 → 110	32	10	5
Theobromine	THB	40	181 → 138	25	10	7	181 → 110	31	10	5
Ferulic acid	FER	45	195 → 177	15	4	10	195 → 145	23	7	7
Syringic acid	SYR	50	199 → 140	21	10	7	199 → 155	15	4	9
(-)-Gallocatechin	GC	45	307 → 139	21	3	12	307 → 151	15	3	8
(+)-Catechin	C	70	291 → 139	20	2	6	291 → 165	17	7	9
(-)-Epicatechin	EC	70	291 → 139	20	2	6	291 → 165	17	7	9
**Negative ionization**										
Carnosol	CAR	-50	329 → 285	-30	-10	-2	329 → 201	-60	-10	-2
Sinapic acid	SIN	-40	223 → 208	-20	-10	-3	223 → 164	-22	-10	-2
Quercetin	QUR	-80	301 → 151	-31	-10	-7	301 → 179	-26	-10	-2
Salicylic acid	SAL	-20	137 → 93	-26	-10	-20	137 → 65	-40	-10	-4
Caffeic acid	CAFAC	-45	179 → 135	-25	-10	-5	179 → 89	-47	-10	-6
p-Coumaric acid	COU	-35	163 → 119	-26	-10	-5	163 → 93	-47	-10	-6

Kaempferol	KAE	-80	285 → 117	-60	-10	-4	285 → 93	-50	-10	-6
Rutin	RUT	-80	609 → 300	-53	-10	-6	609 → 271	-80	-10	-4
Myricetin	MYR	-80	317 → 151	-36	-10	-8	317 → 179	-30	-10	-2
Rosmarinic acid	ROSM	-50	359 → 161	-23	-10	-2	359 → 197	-25	-10	-3
Quinic acid	QUI	-55	191 → 85	-31	-10	-5	191 → 93	-31	-10	-6
Chlorogenic acid	5-CQ	-40	353 → 191	-30	-10	-2	353 → 85	-62	-10	-5
4-O-Caffeoylquinic acid	4-CQ	-40	353 → 191	-30	-10	-2	353 → 85	-62	-10	-5
3-O-Caffeoylquinic acid	3-CQ	-40	353 → 191	-30	-10	-2	353 → 85	-62	-10	-5
3,4-Dicaffeoylquinic acid	3,4-DCQ	-65	515 → 353	-30	-10	-7	515 → 173	-40	-10	-8
3,5-Dicaffeoylquinic acid	3,5-DCQ	-65	515 → 353	-30	-10	-7	515 → 173	-40	-10	-8
4,5-Dicaffeoylquinic acid	4,5-DCQ	-65	515 → 353	-30	-10	-7	515 → 173	-40	-10	-8
(-)-Epicatechin-3-gallate	ECG	-80	441 → 169	-29	-10	-2	441 → 289	-27	-10	-5
(-)-Catechin 3-gallate	CG	-80	441 → 169	-29	-10	-2	441 → 289	-27	-10	-5
(-)-Epigallocatechin 3-gallate	EGCG	-80	457 → 169	-24	-10	-2	457 → 305	-28	-10	-7
(-)-Gallocatechin 3-gallate	GCG	-80	457 → 169	-24	-10	-2	457 → 305	-28	-10	-7
Gallic acid	GAL	-45	169 → 125	-22	-10	-7	169 → 95	-56	-10	-6
Succinic acid	SUC	-31	117 → 73	-17	-10	-4	117 → 99	-16	-10	-7
Protocatechuic acid	PCCA	-45	153 → 109	-22	-10	-5	153 → 91	-37	-10	-3
Carnosic acid	CARAC	-50	331 → 287	-40	-10	-2	331 → 271	-60	-10	-5

[a]DP – declustering potential, [b]CE – collision energy, [c]EP – cell entrance potential, [d]CXP – cell exit potential

Table S3. LC-MS/MS parameters for determined compounds (RT – retention time; LOD – limit of detection; LOQ – limit of quantification)

Compound	RT [min]	Intercept	Slope	R^2	Range [µg L^{-1}]		LOD [µg mL^{-1}]	LOQ [µg mL^{-1}]	Matrix effect
					min	max			
**Positive ionization**									
Melatonin	5.09	5559	71306322	0.9991	0.02	20	0.009	0.029	0.80
Trigonelline	0.79	3076	23244401	0.9996	0.25	5	0.078	0.260	1.02
Nicotinamide	0.88	2241	24552039	0.9997	0.25	5	0.044	0.147	1.11
Nicotinic acid	0.93	4426	20306652	0.9997	0.25	25	0.073	0.242	0.98
Theanine	0.82	3985	18463549	1.0000	0.2	100	0.067	0.221	0.94
Serotonin	1.02	7197	12773909	0.9998	1	100	0.205	0.681	1.01
Theophylline	2.12	5869	17820176	0.9999	0.5	100	0.080	0.267	1.08
Caffeine	2.74	41582	91746500	0.9996	0.1	50	0.018	0.057	0.91
Theobromine	1.53	12456	7083645	0.9995	0.5	500	0.146	0.485	0.95
Ferulic acid	5.13	11086	14287256	1.0000	2.5	250	0.447	1.491	0.90
Syringic acid	3.93	4215	4663320	0.9999	0.5	500	0.143	0.478	1.01
(-)-Gallocatechin	2.47	-475098	4026934	0.9911	100	1000	0.150	0.499	0.81
(+)-Catechin	3.60	-181	3165383	0.9999	10	1000	0.157	0.522	1.16
(-)-Epicatechin	4.02	-18871	3266408	0.9999	10	1000	0.177	0.589	0.89
**Negative ionization**									
Carnosol	7.87	48352	3088414331	0.9993	0.02	2	0.001	0.005	
Sinapic acid	5.13	-33777	21188172	0.9995	5	50	0.050	0.167	0.89
Quercetin	7.19	52508	32001571	0.9997	2	100	0.491	1.637	1.04
Salicylic acid	6.30	58347	36445012	0.9991	2.5	250	0.431	1.435	1.01
Caffeic acid	4.30	54083	9970479	0.9992	5	500	0.106	0.353	1.07
p-Coumaric acid	5.32	-16537	20216685	0.9994	2.5	500	0.299	0.997	0.91

Kaempferol	7.66	15276	7701489	0.9999	1	500	0.039	0.128	0.83
Rutin	4.96	-951	3349114	0.9999	1	500	0.189	0.631	0.85
Myricetin	6.43	-21665	9977608	0.9998	5	500	0.067	0.222	0.87
Rosmarinic acid	5.68	33954	2201296	0.9994	10	1000	2.273	7.576	0.95
Quinic acid	0.83	13535	7406057	0.9999	2	1000	0.300	1,000	1.15
Chlorogenic acid	2.58	7511	2596631	0.9999	5	1000	0.917	3,054	1.12
4-O-Caffeoylquinic acid	3.46	35118	14544079	0.9997	5	500	0.151	0,502	1.14
3-O-Caffeoylquinic acid	3.76	-28912	9998448	0.9999	5	1000	0.504	1,681	1.02
3,4-Dicaffeoylquinic acid	5.36	12073	13497027	1.0000	5	1000	0.321	1,069	1.04
3,5-Dicaffeoylquinic acid	5.53	-228582	2563779	0.9994	100	1000	1.111	3.704	1.02
4,5-Dicaffeoylquinic acid	5.73	998	3382785	0.9999	2.5	500	0.273	0.910	1.16
(-)-Epicatechin-3-gallate	5.17	13350	8969012	0.9984	20	1000	0.134	0.446	1.01
(-)-Catechin 3-gallate	5.41	-994672	9301885	0.9943	100	1000	0.913	3.043	0.85
(-)-Epigallocatechin 3-gallate	4.62	-1022049	11316967	0.9984	100	1000	0.545	1.817	1.07
(-)-Gallocatechin 3-gallate	4.88	-249434	14830206	0.9999	20	500	0.338	1.126	1.06
Gallic acid	1.64	51832	6930818	0.9988	5	500	1.422	4.739	0.99
Succinic acid	1.06	65286	24526779	0.9998	2	500	0.321	1.071	0.84
Protocatechuic acid	6.30	-453	2518596	0.9967	2	100	0.162	0.540	1.04
Carnosic acid	8.43	48352	3088414331	0.9993	0.02	2	0.001	0.005	1.14

Table S4. Oregano extraction optimization test results

Experiment	Parameters		Results		
	Time [min]	Ethanol content [%]	F-C [mg GAE mL^{-1}]	ABTS [mg Trolox mL^{-1}]	DPPH [mg Trolox mL^{-1}]
1	8.7	14.6	1.3306	1.6014	1.4738
2	8.7	85.4	0.7858	1.4561	0.7478
3	26.3	14.6	1.6810	2.0620	2.5120
4	26.3	85.4	1.2166	2.4824	1.2141
5	5	50	1.4161	2.4234	2.2458
6	30	50	1.8499	2.7426	2.2128
7	17.5	0	0.6168	0.7305	0.5383
8	17.5	100	0.4484	1.2358	0.0533
9	17.5	50	2.2078	2.7094	1.7436
10	17.5	50	2.1668	3.2056	2.2910
11	17.5	50	1.9234	2.5052	1.5004
12	17.5	50	1.7485	2.8539	2.4151
13	17.5	50	1.8664	2.8246	1.9818

* – the Folin-Ciocalteu’s method results

Table S5. Sage extraction optimization test results

Experiment	Parameters		Results		
	Time [min]	Ethanol content [%]	F-C [mg GAE mL^{-1}]	ABTS [mg Trolox mL^{-1}]	DPPH [mg Trolox mL^{-1}]
1	8.7	14.6	0.4529	0.6255	0.6442
2	8.7	85.4	0.8677	1.1676	2.9460
3	26.3	14.6	0.9812	1.4278	1.3592
4	26.3	85.4	1.2816	1.7645	2.7219
5	5	50	0.9007	1.1765	1.8999
6	30	50	1.8609	2.5112	2.5819
7	17.5	0	0.1060	0.1640	0.2017
8	17.5	100	0.6203	0.8952	1.2290
9	17.5	50	1.5015	2.0784	2.4602
10	17.5	50	1.5220	2.3089	2.5969
11	17.5	50	1.3331	2.1075	2.0416
12	17.5	50	1.3296	2.1229	2.2983
13	17.5	50	1.6360	2.0549	2.4038

* – the Folin-Ciocalteu’s method results

Table S6. Basil extraction optimization test results

Experiment	Parameters		Results		
	Time [min]	Ethanol content [%]	F-C [mg GAE mL^{-1}]	ABTS [mg Trolox mL^{-1}]	DPPH [mg Trolox mL^{-1}]
1	8.7	14.6	1.4371	1.8657	2.2493
2	8.7	85.4	0.4986	0.5380	0.5662
3	26.3	14.6	1.4846	2.1260	2.6106
4	26.3	85.4	0.5078	0.5764	0.5270
5	5	50	0.9890	1.1938	1.4205
6	30	50	1.6725	2.3059	2.9720
7	17.5	0	1.5750	1.9389	2.0334
8	17.5	100	0.0107	0.0921	0.1766
9	17.5	50	1.3441	1.8369	2.3663
10	17.5	50	1.3136	1.8620	1.8880
11	17.5	50	1.2169	1.6918	1.7796
12	17.5	50	1.0252	1.4613	1.5332
13	17.5	50	1.5043	1.5944	1.5935

* – the Folin-Ciocalteu's method results

Table S7. Rosemary extraction optimization test results

Experiment	Parameters		Results		
	Time [min]	Ethanol content [%]	F-C [mg GAE mL^{-1}]	ABTS [mg Trolox mL^{-1}]	DPPH [mg Trolox mL^{-1}]
1	8.7	14.6	0.3181	0.3784	0.4072
2	8.7	85.4	0.3359	0.4109	0.4966
3	26.3	14.6	0.4687	0.6247	0.8018
4	26.3	85.4	0.6828	0.7526	0.9811
5	5	50	0.4086	0.5247	0.5681
6	30	50	0.6484	0.8454	1.0560
7	17.5	0	0.3922	0.4717	0.4982
8	17.5	100	0.2595	0.1941	0.4428
9	17.5	50	0.6331	0.8425	1.0439
10	17.5	50	0.7051	0.9660	1.1342
11	17.5	50	0.4390	0.6210	0.7212
12	17.5	50	0.5330	0.6858	0.8213
13	17.5	50	0.6472	0.7581	0.8475

* – the Folin-Ciocalteu's method results

Table S8. The response surfaces observed in the tests Folin-Ciocalteu, ABTS, and DPPH for herbs

The response surfaces in the tests		
**Folin-Ciocalteu**	**ABTS**	**DPPH**
**Oregano**		
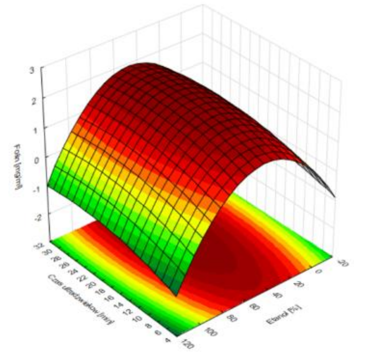	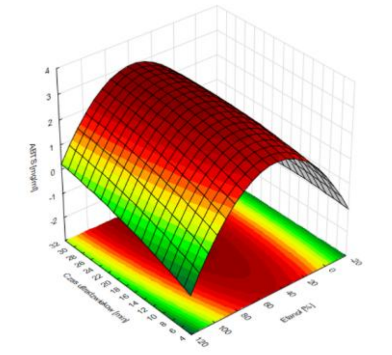	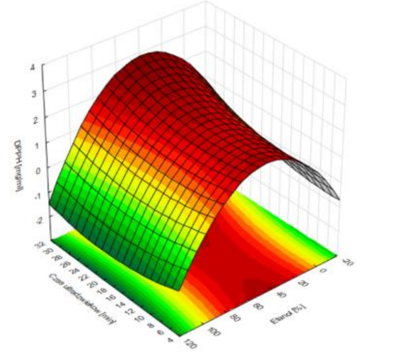
**Sage**		
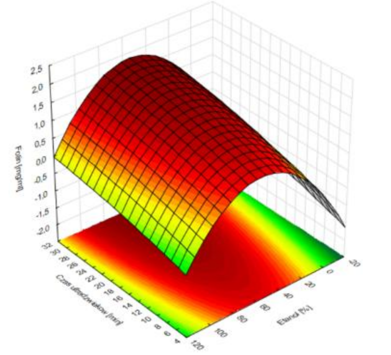	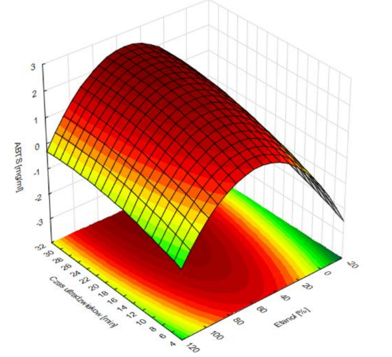	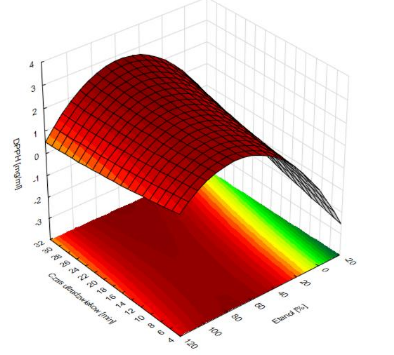
**Basil**		
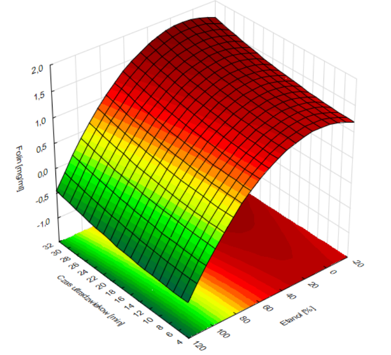	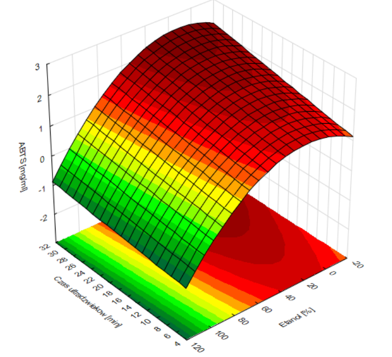	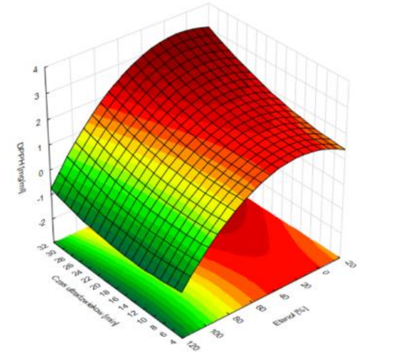
**Rosemary**		
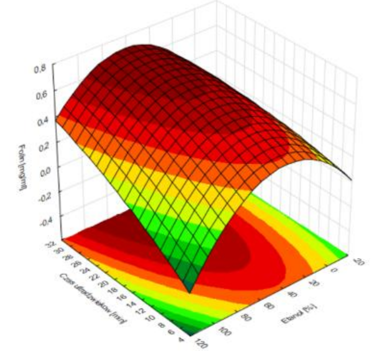	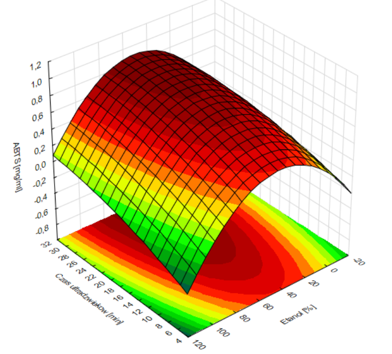	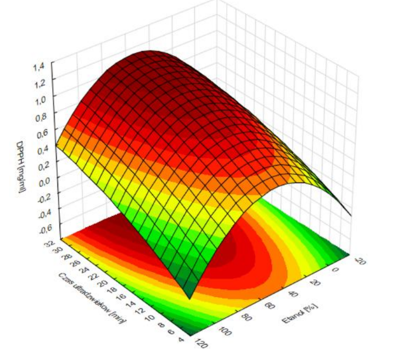

Table S9. Pareto charts in the tests Folin-Ciocalteu, ABTS, and DPPH for herbs

The response surfaces in the tests		
**Folin-Ciocalteu**	**ABTS**	**DPPH**
	**Oregano**	
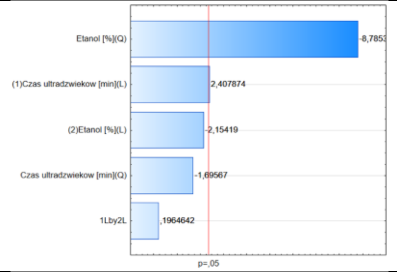	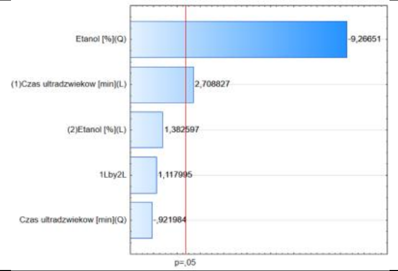	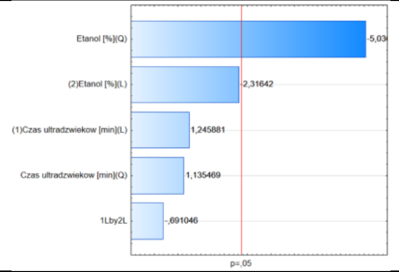
	**Sage**	
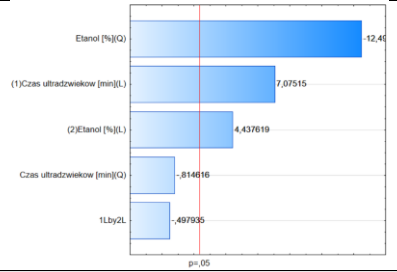	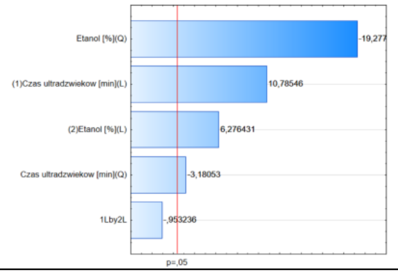	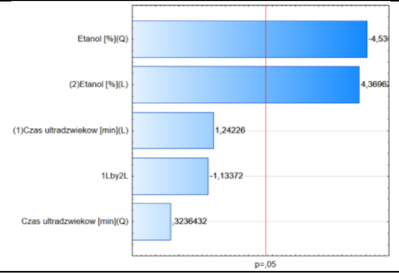
	**Basil**	
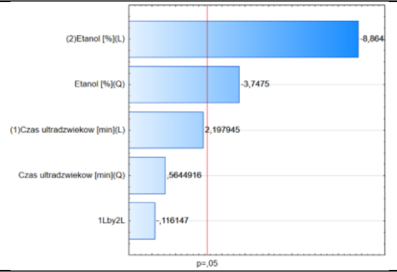	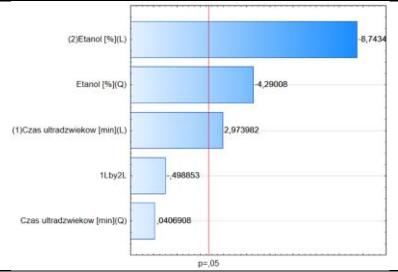	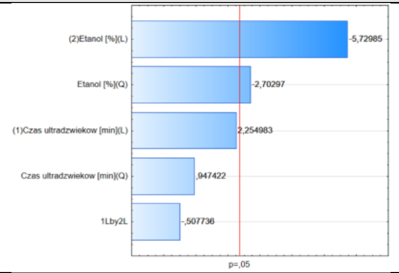
	**Rosemary**	
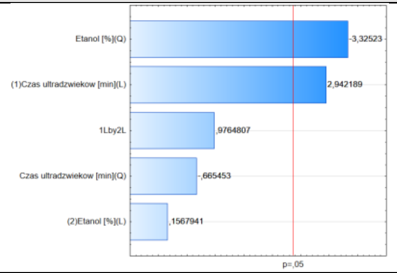	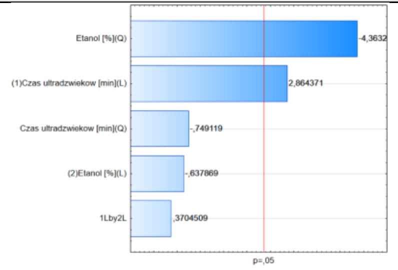	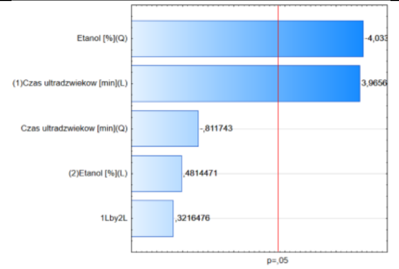

Table S10. Optimization of oregano extraction towards the greatest total phenolic content determined by the Folin-Ciocalteu test (R^2: 0.9263, Adjusted R^2: 0.8736, Predicted R^2: 0.6940, C.V. %: 13.83, Adequate Precision: 11.384; $y = 0.034422 + (0.075491)\cdot A + (0.049007)\cdot B + (-0.001685)\cdot A^2 + (-0.000545)\cdot B^2 + (0.000064)\cdot AB$)

Source	Sum of Squares	df	Mean Square	F-value	p-value Prob > F
**Model**	**3.6902**	**5**	**0.7380**	**17.59**	**0.0008 ***
A - Sonication time	0.1548	1	0.1548	3.69	0.0962
B - Ethanol content	1.3339	1	1.3339	31.80	0.0008 *
A^2	0.1206	1	0.1206	2.88	0.1338
B^2	3.2376	1	3.2376	77.18	< 0.0001 **
AB	0.0016	1	0.0016	0.04	0.8498
Residual	0.2936	7	0.0419	-	-
Lack of Fit	0.1371	3	0.0457	1.17	0.4256
Pure Error	0.1565	4	0.0391	-	-
**Cor Total**	**3.9839**	**12**	**-**	**-**	**-**

Table S11. Optimization of oregano extraction towards the greatest antioxidant activity determined by the ABTS assay (R^2: 0.9323, Adjusted R^2: 0.8840, Predicted R^2: 0.7375, C.V. %: 11.41, Adequate Precision: 12.318; $y = 0.437440 + (0.044429)\cdot A + (0.066649)\cdot B + (-0.001132)\cdot A^2 + (-0.000711)\cdot B^2 + (0.000452)\cdot AB$)

Source	Sum of Squares	df	Mean Square	F-value	p-value Prob > F
**Model**	**6.1731**	**5**	**1.2346**	**19.29**	**0.0006 ***
A - Sonication time	0.0536	1	0.0536	0.84	0.3906
B - Ethanol content	2.4672	1	2.4672	38.55	0.0004 *
A^2	0.0544	1	0.0544	0.85	0.3872
B^2	5.4960	1	5.4960	85.87	< 0.0001 **
AB	0.0800	1	0.0800	1.25	0.3005
Residual	0.4480	7	0.0640	-	-
Lack of Fit	0.1869	3	0.0623	0.95	0.4952
Pure Error	0.2612	4	0.0653	-	-
**Cor Total**	**6.6212**	**12**	**-**	**-**	**-**

Table S12. Optimization of oregano extraction towards the greatest antioxidant activity determined by the DPPH assay (R^2: 0.8370, Adjusted R^2: 0.7206, Predicted R^2: 0.2725, C.V. %: 25.70, Adequate Precision: 9.499; $y = 0.825632 + (-0.036325)\cdot A + (0.061500)\cdot B + (0.002279)\cdot A^2 + (-0.000631)\cdot B^2 + (-0.000457)\cdot AB$)

Source	Sum of Squares	df	Mean Square	F-value	p-value Prob > F
**Model**	**6.1537**	**5**	**1.2307**	**7.19**	**0.0111 ***
A - Sonication time	0.0358	1	0.0358	0.21	0.6612
B - Ethanol content	2.1007	1	2.1007	12.27	0.0099 *
A^2	0.2207	1	0.2207	1.29	0.2936
B^2	4.3311	1	4.3311	25.30	0.0015 *
AB	0.0817	1	0.0817	0.48	0.5118
Residual	1.1982	7	0.1712	-	-
Lack of Fit	0.6264	3	0.2088	1.46	0.3514
Pure Error	0.5718	4	0.1429	-	-
**Cor Total**	**7.3519**	**12**	**-**	**-**	**-**

Table S13. Optimization of sage extraction towards the greatest total phenolic content determined by the Folin-Ciocalteu test (R^2: 0.9701, Adjusted R^2: 0.9487, Predicted R^2: 0.9120, C.V. %: 10.38, Adequate Precision: 21.503; $y = -0.667450 + (0.053001)\cdot A + (0.050244)\cdot B + (-0.000454)\cdot A^2 + (-0.000435)\cdot B^2 + (-0.000091)\cdot AB$)

Source	Sum of Squares	df	Mean Square	F-value	p-value Prob > F
**Model**	**2.9973**	**5**	**0.5995**	**45.38**	**< 0.0001 ****
A - Sonication time	0.0763	1	0.0763	5.77	0.0473 *
B - Ethanol content	1.4021	1	1.4021	106.13	< 0.0001 **
A^2	0.0088	1	0.0088	0.66	0.4421
B^2	2.0638	1	2.0638	156.22	< 0.0001 **
AB	0.0033	1	0.0033	0.25	0.6338
Residual	0.0925	7	0.0132	-	-
Lack of Fit	0.0229	3	0.0076	0.44	0.7375
Pure Error	0.0696	4	0.0174	-	-
**Cor Total**	**3.0898**	**12**	**-**	**-**	**-**

Table S14. Optimization of sage extraction towards the greatest antioxidant activity determined by the ABTS assay (R^2: 0.9869, Adjusted R^2: 0.9776, Predicted R^2: 0.9434, C.V. %: 6.86, Adequate Precision: 30.508; $y = -1.243599 + (0.112881)\cdot A + (0.072589)\cdot B + (-0.001663)\cdot A^2 + (-0.000630)\cdot B^2 + (-0.000164)\cdot AB$)

Source	Sum of Squares	df	Mean Square	F-value	p-value Prob > F
**Model**	**6.1377**	**5**	**1.2275**	**105.75**	**< 0.0001 ****
A - Sonication time	0.3460	1	0.3460	29.81	0.0009 *
B - Ethanol content	2.9265	1	2.9265	252.10	< 0.0001 **
A^2	0.1174	1	0.1174	10.12	0.0155 *
B^2	4.3141	1	4.3141	371.64	< 0.0001 **
AB	0.0105	1	0.0105	0.91	0.3722
Residual	0.0813	7	0.0116	-	-
Lack of Fit	0.0405	3	0.0135	1.33	0.3833
Pure Error	0.0408	4	0.0102	-	-
**Cor Total**	**6.2190**	**12**	**-**	**-**	**-**

Table S15. Optimization of sage extraction towards the greatest antioxidant activity determined by the DPPH assay (R^2: 0.8609, Adjusted R^2: 0.7616, Predicted R^2: 0.1229, C.V. %: 21.21, Adequate Precision: 9.548; $y = -0.785222 + (0.035326)\cdot A + (0.088152)\cdot B + (0.000650)\cdot A^2 + (-0.000569)\cdot B^2 + (-0.000750)\cdot AB$)

Source	Sum of Squares	df	Mean Square	F-value	p-value Prob > F
**Model**	**7.4338**	**5**	**1.4868**	**8.67**	**0.0066 ***
A - Sonication time	0.0339	1	0.0339	0.20	0.6701
B - Ethanol content	4.3158	1	4.3158	25.16	0.0015 *
A^2	0.0180	1	0.0180	0.10	0.7557
B^2	3.5281	1	3.5281	20.57	0.0027 *
AB	0.2205	1	0.2205	1.29	0.2942
Residual	1.2008	7	0.1715	-	-
Lack of Fit	1.0276	3	0.3425	7.91	0.0371 *
Pure Error	0.1732	4	0.0433	-	-
**Cor Total**	**8.6346**	**12**	**-**	**-**	**-**

Table S16. Optimization of basil extraction towards the greatest total phenolic content determined by the Folin-Ciocalteu test (R^2: 0.9337, Adjusted R^2: 0.8864, Predicted R^2: 0.6493, C.V. %: 14.99, Adequate Precision: 14.765; $y = 1.340451 + (0.000218)\cdot A + (0.004651)\cdot B + (0.000451)\cdot A^2 + (-0.000187)\cdot B^2 + (-0.000031)\cdot AB$)

Source	Sum of Squares	df	Mean Square	F-value	p-value Prob > F
**Model**	**2.6715**	**5**	**0.5343**	**19.72**	**0.0005 ***
A - Sonication time	0.0000	1	0.0000	0.00	0.9947
B - Ethanol content	0.0120	1	0.0120	0.44	0.5268
A^2	0.0086	1	0.0086	0.32	0.5900
B^2	0.3805	1	0.3805	14.04	0.0072 *
AB	0.0004	1	0.0004	0.01	0.9108
Residual	0.1897	7	0.0271	-	-
Lack of Fit	0.1273	3	0.0424	2.72	0.1790
Pure Error	0.0624	4	0.0156	-	-
**Cor Total**	**2.8612**	**12**	**-**	**-**	**-**

Table S17. Optimization of basil extraction towards the greatest antioxidant activity determined by the ABTS assay (R^2: 0.9371, Adjusted R^2: 0.8922, Predicted R^2: 0.6659, C.V. %: 15.15, Adequate Precision: 15.308; $y = 1.333213 + (0.033791)\cdot A + (0.012594)\cdot B + (0.000044)\cdot A^2 + (-0.000289)\cdot B^2 + (-0.000177)\cdot AB$)

Source	Sum of Squares	df	Mean Square	F-value	p-value Prob > F
**Model**	**5.1625**	**5**	**1.0325**	**20.86**	**0.0004 ***
A - Sonication time	0.0310	1	0.0310	0.63	0.4546
B - Ethanol content	0.0881	1	0.0881	1.78	0.2239
A^2	0.0001	1	0.0001	0.00	0.9687
B^2	0.9109	1	0.9109	18.40	0.0036 *
AB	0.0123	1	0.0123	0.25	0.6332
Residual	0.3464	7	0.0495	-	-
Lack of Fit	0.2338	3	0.0779	2.77	0.1751
Pure Error	0.1126	4	0.0282	-	-
**Cor Total**	**5.5089**	**12**	**-**	**-**	**-**

Table S18. Optimization of basil extraction towards the greatest antioxidant activity determined by the DPPH assay (R^2: 0.8708, Adjusted R^2: 0.7786, Predicted R^2: 0.3691, C.V. %: 23.62, Adequate Precision: 10.778; $y = 1.806362 + (-0.011892)\cdot A + (0.015333)\cdot B + (0.001814)\cdot A^2 + (-0.000323)\cdot B^2 + (-0.000320)\cdot AB$)

Source	Sum of Squares	df	Mean Square	F-value	p-value Prob > F
**Model**	**7.3444**	**5**	**1.4689**	**9.44**	**0.0051 ***
A - Sonication time	0.0038	1	0.0038	0.02	0.8796
B - Ethanol content	0.1306	1	0.1306	0.84	0.3901
A^2	0.1397	1	0.1397	0.90	0.3750
B^2	1.1370	1	1.1370	7.31	0.0305 *
AB	0.0401	1	0.0401	0.26	0.6272
Residual	1.0894	7	0.1556	-	-
Lack of Fit	0.6518	3	0.2173	1.99	0.2583
Pure Error	0.4376	4	0.1094	-	-
**Cor Total**	**8.4338**	**12**	**-**	**-**	**-**

Table S19. Optimization of rosemary extraction towards the greatest total phenolic content determined by the Folin-Ciocalteu test (R^2: 0.7477; Adjusted R^2: 0.5675; Predicted R^2: 0.0854; C.V. %: 20.20; Adequate Precision: 5.822; $y = 0.161047 + (0.015350)\cdot A + (0.007546)\cdot B + (-0.000325)\cdot A^2 + (-0.000101)\cdot B^2 + (0.000157)\cdot AB$)

Source	Sum of Squares	df	Mean Square	F-value	p-value Prob > F
**Model**	**0.2097**	**5**	**0.0419**	**4.15**	**0.0452 ***
A - Sonication time	0.0064	1	0.0064	0.63	0.4524
B - Ethanol content	0.0316	1	0.0316	3.13	0.1202
A^2	0.0045	1	0.0045	0.44	0.5271
B^2	0.1118	1	0.1118	11.06	0.0127 *
AB	0.0096	1	0.0096	0.95	0.3614
Residual	0.0708	7	0.0101	-	-
Lack of Fit	0.0263	3	0.0088	0.79	0.5592
Pure Error	0.0444	4	0.0111	-	-
**Cor Total**	**0.2805**	**12**	**-**	**-**	**-**

Table S20. Optimization of rosemary extraction towards the greatest antioxidant activity determined by the ABTS assay (R^2: 0.7990, Adjusted R^2: 0.6554, Predicted R^2: 0.2746, C.V. %: 20.70, Adequate Precision: 6.608; $y = 0.056221 + (0.027282) \cdot A + (0.014853) \cdot B + (-0.000467) \cdot A^2 + (-0.000170) \cdot B^2 + (0.000076) \cdot AB$)

Source	Sum of Squares	df	Mean Square	F-value	p-value Prob > F
**Model**	**0.4599**	**5**	**0.0920**	**5.56**	**0.0219 ***
A - Sonication time	0.0202	1	0.0202	1.22	0.3054
B - Ethanol content	0.1225	1	0.1225	7.41	0.0297 *
A^2	0.0093	1	0.0093	0.56	0.4782
B^2	0.3147	1	0.3147	19.04	0.0033 *
AB	0.0023	1	0.0023	0.14	0.7220
Residual	0.1157	7	0.0165	-	-
Lack of Fit	0.0427	3	0.0142	0.78	0.5634
Pure Error	0.0730	4	0.0182	-	-
**Cor Total**	**0.5756**	**12**	**-**	**-**	**-**

Table S21. Optimization of rosemary extraction towards the greatest antioxidant activity determined by the DPPH assay (R^2: 0.8224, Adjusted R^2: 0.6955, Predicted R^2: 0.5685, C.V. %: 18.52, Adequate Precision: 6.865; $y = -0.041776 + (0.037881) \cdot A + (0.016518) \cdot B + (-0.000551) \cdot A^2 + (-0.000171) \cdot B^2 + (0.000072) \cdot AB$)

Source	Sum of Squares	df	Mean Square	F-value	p-value Prob > F
**Model**	**0.6343**	**5**	**0.1269**	**6.48**	**0.0147 ***
A - Sonication time	0.0390	1	0.0390	1.99	0.2011
B - Ethanol content	0.1515	1	0.1515	7.74	0.0272 *
A^2	0.0129	1	0.0129	0.66	0.4437
B^2	0.3183	1	0.3183	16.27	0.0050 *
AB	0.0020	1	0.0020	0.10	0.7571
Residual	0.1370	7	0.0196	-	-
Lack of Fit	0.0214	3	0.0071	0.25	0.8599
Pure Error	0.1156	4	0.0289	-	-
**Cor Total**	**0.7713**	**12**	**-**	**-**	**-**

Table S22. Extraction parameters obtained for herbs

Extraction parameters	F-C	ABTS	DPPH	Optimal	Used
**Oregano**					
US [min]	23.3	30.0	30.0	27.8	**30**
EtOH [%]	46.3	56.4	37.9	46.9	**50**
**Sage**					
US [min]	30.0	30.0	30.0	30.0	**30**
EtOH [%]	54.6	53.8	57.7	55.4	**55**
**Basil**					
US [min]	30.0	30.0	30.0	30.0	**30**
EtOH [%]	9.9	12.6	8.9	10.5	**10**
**Rosemary**					
US [min]	30.0	30.0	30.0	30.0	**30**
EtOH [%]	60.7	50.4	54.6	55.2	**55**

Table S23. Spearman's correlation coefficients meaning

Correlation range	Meaning
$\|r\| = 0.00 – 0.10$	Negligible correlation
$\|r\| = 0.10 – 0.39$	Weak correlation
$\|r\| = 0.40 – 0.69$	Moderate correlation
$\|r\| = 0.70 – 0.89$	Strong correlation
$\|r\| = 0.90 – 1.00$	Very strong correlation

Table S24. Spearman's correlation between the content of rosmarinic acid (ROSM) and the total phenolic content (the Folin-Ciocalteu test results)

Sample	ROSM [µg/g]	Folin-Ciocalteu [mg GAE / mL]	Rank X	Rank Y
O1	3645	2.3873	6	17
O2	7861	2.4453	15	18
O3	5962	2.2868	10	15
O4	9801	3.7304	20	22
O5	5771	2.2598	9	14
S1	7722	2.4463	14	19
S2	15556	2.3148	22	16
S3	1041	0.6708	1	1
B1	6731	1.6623	12	9
B2	3510	1.5505	5	6
B3	6540	2.2568	11	13
B4	8158	2.2191	16	12
B5	8181	2.0364	17	11
R1	1121	1.1033	2	2
R2	2622	1.1141	4	4
R3	9581	1.3710	19	5
R4	1504	1.1057	3	3
HP1	4625	1.6035	7	7
HP2	9334	2.8317	18	21
HP3	10255	2.5189	21	20
DH1	7684	1.9874	13	10
SH1	5491	1.6575	8	8
**Spearman's correlation coefficient**			**0.7030**	
p-value			0.0003	

Table S25. Spearman's correlation between the content of rosmarinic acid (ROSM) and the antioxidant activity (ABTS assay results)

Sample	ROSM [μg/g]	ABTS [mg Trolox / mL]	Rank X	Rank Y
O1	3645	3.9097	6	15
O2	7861	4.3126	15	19
O3	5962	3.9937	10	16
O4	9801	6.1835	20	22
O5	5771	4.3646	9	20
S1	7722	3.4981	14	11
S2	15556	3.5494	22	13
S3	1041	1.1762	1	1
B1	6731	2.2183	12	7
B2	3510	2.1733	5	6
B3	6540	3.5415	11	12
B4	8158	3.5893	16	14
B5	8181	4.0403	17	17
R1	1121	1.4131	2	3
R2	2622	1.3531	4	2
R3	9581	1.6578	19	5
R4	1504	1.6452	3	4
HP1	4625	2.2452	7	8
HP2	9334	4.0635	18	18
HP3	10255	4.5919	21	21
DH1	7684	2.9268	13	10
SH1	5491	2.8712	8	9
**Spearman's correlation coefficient**			**0.6657**	
p-value			0.0007	

Table S26. Spearman's correlation between the content of rosmarinic acid (ROSM) and the antioxidant activity (DPPH assay results)

Sample	ROSM [μg/g]	DPPH [mg Trolox / mL]	Rank X	Rank Y
O1	3645	2.4029	6	13
O2	7861	2.6867	15	15
O3	5962	2.3159	10	11
O4	9801	6.7007	20	22
O5	5771	2.3672	9	12
S1	7722	3.3675	14	20
S2	15556	3.4000	22	21
S3	1041	0.9356	1	1
B1	6731	2.2694	12	10
B2	3510	2.1245	5	8
B3	6540	3.3170	11	18
B4	8158	3.1457	16	16
B5	8181	3.2891	17	17
R1	1121	1.3831	2	5
R2	2622	1.3214	4	4
R3	9581	1.2465	19	3
R4	1504	1.2362	3	2
HP1	4625	2.0065	7	7
HP2	9334	3.3628	18	19
HP3	10255	2.6072	21	14
DH1	7684	2.1926	13	9
SH1	5491	1.5100	8	6
**Spearman's correlation coefficient**			**0.7188**	
p-value			0.0002	

Table S27. Spearman's correlation between the content of quinic acid (QUI) and the total phenolic content (the Folin-Ciocalteu test results)

**Sample**	**QUI [µg/g]**	**Folin-Ciocalteu [mg GAE / mL]**	**Rank X**	**Rank Y**
O1	7885	2.3873	22	17
O2	6265	2.4453	18	18
O3	7136	2.2868	20	15
O4	4078	3.7304	14	22
O5	6358	2.2598	19	14
S1	19	2.4463	1	19
S2	25	2.3148	2	16
S3	65	0.6708	3	1
B1	1451	1.6623	6	9
B2	1603	1.5505	9	6
B3	1629	2.2568	10	13
B4	1573	2.2191	8	12
B5	877	2.0364	4	11
R1	3088	1.1033	11	2
R2	1406	1.1141	5	4
R3	6150	1.3710	17	5
R4	5973	1.1057	16	3
HP1	1481	1.6035	7	7
HP2	3710	2.8317	13	21
HP3	5240	2.5189	15	20
DH1	3395	1.9874	12	10
SH1	7307	1.6575	21	8
**Spearman's correlation coefficient**			**0.2027**	
p-value			0.3656	

Table S28. Spearman's correlation between the content of quinic acid (QUI) and the antioxidant activity (ABTS assay results)

Sample	QUI [μg/g]	ABTS [mg Trolox / mL]	Rank X	Rank Y
O1	7885	3.9097	22	15
O2	6265	4.3126	18	19
O3	7136	3.9937	20	16
O4	4078	6.1835	14	22
O5	6358	4.3646	19	20
S1	19	3.4981	1	11
S2	25	3.5494	2	13
S3	65	1.1762	3	1
B1	1451	2.2183	6	7
B2	1603	2.1733	9	6
B3	1629	3.5415	10	12
B4	1573	3.5893	8	14
B5	877	4.0403	4	17
R1	3088	1.4131	11	3
R2	1406	1.3531	5	2
R3	6150	1.6578	17	5
R4	5973	1.6452	16	4
HP1	1481	2.2452	7	8
HP2	3710	4.0635	13	18
HP3	5240	4.5919	15	21
DH1	3395	2.9268	12	10
SH1	7307	2.8712	21	9
**Spearman's correlation coefficient**			**0.3529**	
p-value			0.1072	

Table S29. Spearman's correlation between the content of quinic acid (QUI) and the antioxidant activity (DPPH assay results)

Sample	QUI [μg/g]	DPPH [mg Trolox / mL]	Rank X	Rank Y
O1	7885	2.4029	22	13
O2	6265	2.6867	18	15
O3	7136	2.3159	20	11
O4	4078	6.7007	14	22
O5	6358	2.3672	19	12
S1	19	3.3675	1	20
S2	25	3.4000	2	21
S3	65	0.9356	3	1
B1	1451	2.2694	6	10
B2	1603	2.1245	9	8
B3	1629	3.3170	10	18
B4	1573	3.1457	8	16
B5	877	3.2891	4	17
R1	3088	1.3831	11	5
R2	1406	1.3214	5	4
R3	6150	1.2465	17	3
R4	5973	1.2362	16	2
HP1	1481	2.0065	7	7
HP2	3710	3.3628	13	19
HP3	5240	2.6072	15	14
DH1	3395	2.1926	12	9
SH1	7307	1.5100	21	6
**Spearman's correlation coefficient**			**-0.1429**	
p-value			0.5259	

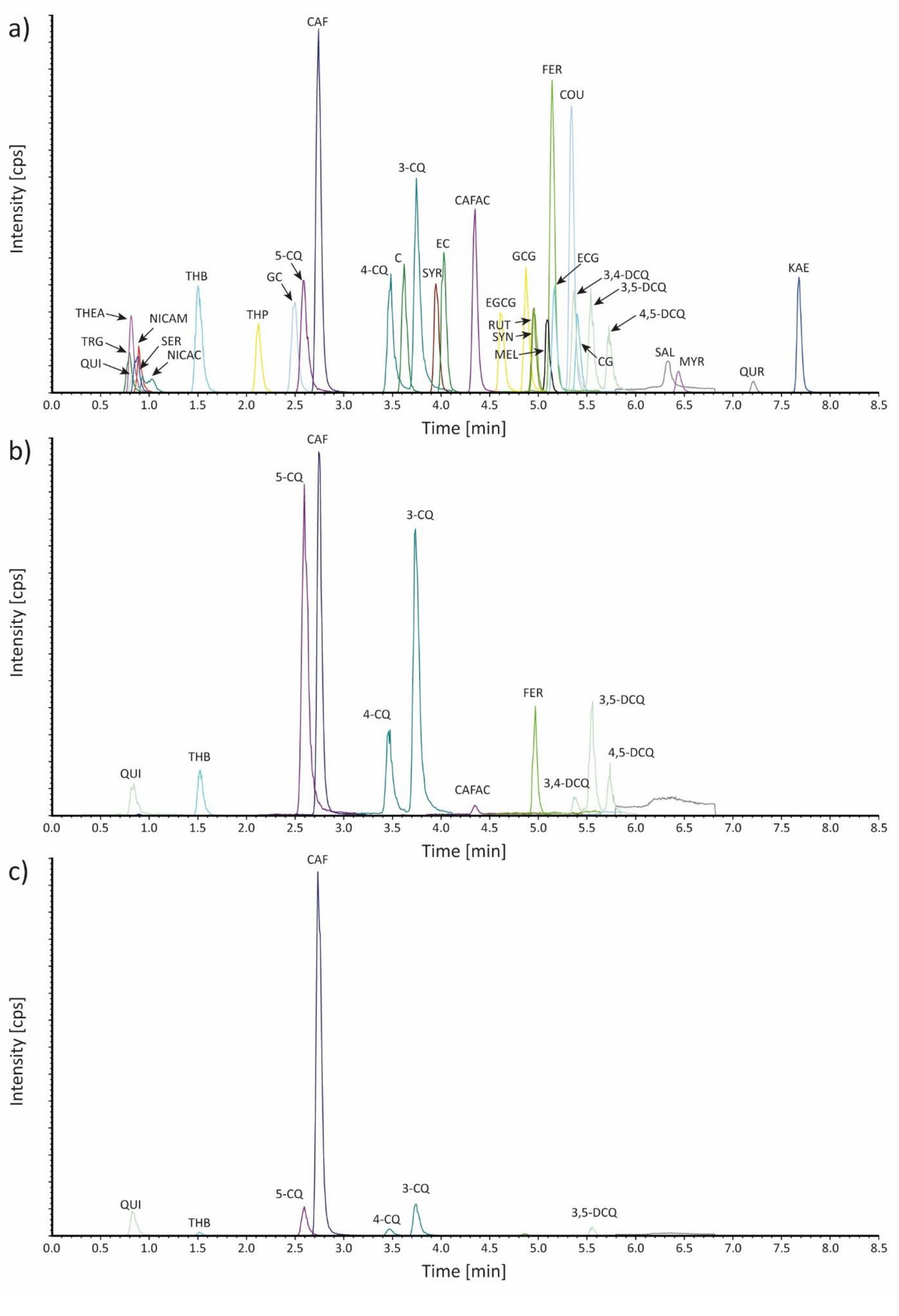

Figure S1. Chromatograms of a) standard solution, b) sample 10, c) sample 8
